# The adjustment of China endemic *Heptacodium miconioides* Rehd. to temperate zone of Poland

**DOI:** 10.1186/s12870-023-04205-y

**Published:** 2023-04-06

**Authors:** Marta Joanna Monder, Przemysław Bąbelewski, Jakub Szperlik, Agnieszka Kościelak

**Affiliations:** 1 Section of Ornamental Plants, Institute of Horticultural Sciences, Warsaw University of Life Sciences (SGGW), 166 Nowoursynowska Str., Warsaw, 02-787 Poland; 2 Department of Horticulture, Wrocław University of Environmental and Life Sciences, Pl. Grunwaldzki 24A, Wrocław, 50-363 Poland; 3 Faculty of Biological Sciences, Botanical Garden, University of Wrocław, 23 Sienkiewicza Str., Wrocław, 50-525 Poland; 4 Department of Dendrological Collections, Polish Academy of Sciences Botanical Garden—Center for Biological Diversity Conservation in Powsin, Prawdziwka 2 Str., Warsaw, 02-973 Poland

**Keywords:** Biodiversity, Cambium, Climate change, Cortex, Dormancy period, Flowering, Phenology, Phloem, Xylem

## Abstract

*Heptacodium miconioides* is an increasingly popular ornamental plant, originally being endemic to China. The late and long flowering determines its ecological and ornamental value in cultivation. The aims of this research were to define and distinguish phenological phases of the development of *Heptacodium miconioides* in the temperate zone region and identification of anatomical changes within the stem during autumn in relation to phenological phases and climatic conditions. Phenological observations were carried out in Wrocław during 2012–2013, as well as in Warsaw (Poland, 52.6°N, 20.5°E) during 2018–2021. During the last year of research an analysis of the anatomical structure was carried out for young stems that bore flowers that year, as well as older, 2- to 6-year-old ones. The material was collected H1 – 10.09., H2 – 28.09., H3 – 16.10., H4 – 3.11., H5 – 21.11. The width of annual increments in subsequent years was determined; length, width and vessel density in early and latewood for subsequent rings of annual growth was measured, as well as the width of the phloem in 1–6-year-old stems (2016–2021). In the vegetative stage three main stages of development were distinguished (leaf buds have the green tips; full autumn discoloration of leaves; leaves falling). In the generative phase, which lasted on average from August 22^nd^ to January the 7^th^ five main phases of development were distinguished (flowering, unripe fruits, ripe fruits, spreading of seeds). Increased average temperature during winter and spring had an effect on the growth pattern: early phenological stages occurred sooner and foliage development lasted 44 days longer. Flowering occurred at a similar date at both observed locations and climatic conditions. This year’s shoots flowering on a radial section with axial symmetry, were slightly flattened and in clusters arranged regularly to match the shape. *Heptacodium* develops 2–6 years old shoots with radial symmetry. The growth ring boundaries are distinct, the wood semi-rings porous, with marked differences in the structure of the primary and secondary shoot. Lignification of tissues before winter ends during late leaf-fall phase. The research indicated the adaptive potential of *Heptacodium* in response to climatic conditions of temperate zone.

## Introduction

*Heptacodium miconioides* Rehder is a representative of the monotypic genus *Heptacodium* of *Caprifoliaceae* family [1]. This species was found in 1907 in Hubei province in China by Ernest Henry Wilson and described by Alfred Rehder (Arnold’s Arboretum, USA) in 1916. *Heptacodium miconioides* is a large shrub or small multi trunked tree, growing to 4–9 m [2].

*Heptacodium miconioides* is rare species [3] endemic to China [1, 4] and has been most recently evaluated for the IUCN Red List of Threatened Species in 1998. Then it was categorized as Vulnerable under criteria A1cd, but the current population trends were unspecified. Its geographic range was given as China (Anhui, Zhejiang, Hubei); however the previously described locations were not confirmed in Hubei and on the remaining ones the plants were found in small numbers only. Population declines due to indiscriminate cutting have been recorded. The most serious threats were ecosystem conversion and degradation, animal husbandry, agriculture and wood harvesting. No CITES legislation for this species was found [5]. The research in reproduction biology indicated that the seed variability in *H. miconioides* was limited by restricted set of sites of natural habitation [6]. There is only limited information on the ecological conditions of the sites *H. miconioides* grows at. The research by [7] indicated, that *H. miconioides* communities in the Tiantai Mountains of Zhejiang Province were at the declining stage and were observed in clump. The vertical structure showed layers split into arborous, shrub, herbaous and interstratum plants with comparatively high diversity index [7, 8]. The analysis showed that the age-class in the majority of dominant populations of the community was incomplete and the age structure was declining. The community was unstable and the distributions were clumping [9]. The random amplified polymorphic DNA (RAPD) technique performed on natural populations of *H. miconioides* of Zhejiang Province showed low gene flow and a distinct genetic differentiation among populations. The genetic differentiation among evaluated nine populations was relatively high, but that within populations was relatively low [10].

*Heptacodium miconioides* is a relatively poorly researched species. Ethanol and acetone extracts from leaves and flowers were shown to have antimicrobial properties on 4 experimental strains, such as *Staphylococcus aureus, Bacillus thuringiensis, Pseudomonas aeruginosa* and *Escherichia coli* [11, 12]. It is worth noting that those properties varied depending on the exact time when the leaves were collected [11, 13]. It was also showed that the changes in content of secondary metabolites (flavonoids, tannins, alkaloids, saponins, lignins and chlorogenic acids) in leaves differed according to the part of the arborous layer [11]. In the available literature only one work briefly discussing the anatomy of this species’ stems in relation to other *Caprifoliacecae* family species was found [14].

Since 1980s *Heptacodium miconioides* grew in popularity in cultivation and is increasingly often planted as an ornamental plant and present in the nursery market. The species is appreciated for its late flowering period (September). The horticultural practice reports indicated a broad tolerance of habitat conditions and soil fertility. Moreover, the scented, nectar-rich flowers attract bees, monarch butterflies and other pollinators. The vivid colors of cherry red to rose-purple fruits, discolored leaves and exfoliating bark add to the value as an ornamental plant in autumn and winter and to the landscape [2, 15]. Cultivation of species with a potentially wide range of tolerance in terms of the cultivation location is in line with the current trends in the selection of species capable of adaptation in response to changing climatic conditions [16].

*Heptacodium miconioides* Rehd. is characterized by a late flowering period [6]. During the flowering period intense morphological and physiological changes take place in the plants, while genes play a crucial role in the formation of flowers, indicating that reproductive development in plants is genetically controlled. Species that flower late probably possess genetically regulated autonomous pathway for floral induction. Environmental factors also play a role, as well as endogenous factors, e.g., carbohydrates (nutrients), hormones, and circadian rhythm [17]. Research on the cultivars of roses has shown that during the short flowering period physiological [18] and anatomical [19] changes take place in the stems from current growing season. Those changes are a decisive factor for the process of rhizogenesis in seedlings [19] and their response to rooting promoting preparations [20, 21].

For species of the temperate zone, it is important to enter the winter dormancy, preceded by the process of woody tissue lignification. Due to this the meristem activity starts early in the spring (March) and finishes by mid-Summer (August) [22]. However, the described proceses do not have to proceed in this way. In some species, cambium activity could be detected even in late autumn. In the case of *Castanea sativa* cambium activity started on 17 April. The last cells on late wood were formed until mid-October and the phloem ring was completed by the beginning of October [23]. The seasonality of cambium, the dynamics of phloem and wood formation, cell size variation, lignification and phenology are often the subject of studies and monitoring of trees in relation to climatic factors [23, 24]. Understanding these processes allows for a deeper examination of the possibilities of adaptation to climate change and preservation of broadly understood biodiversity of living organisms [25].

Processes of xylogenesis is limited by climatic conditions, however there is a relative significance of the endogenous and environmental factors excluding climatic conditions occurring throughout the growing season that affect the cambium activity and definitive phases of xylogenesis [26]. However, if the period of xylem development corresponds to period in which trees and their wood cells are open to receive environmental signals directly [27], then the activity of cambium of late flowering species may delay their induction of entering the dormancy stage. Proper conduct and termination of lignification are usually completed before the winter dormancy in temperate zone, albeit it is not always the case [28]. Differences in timing and dynamics of vessel formation, as well as the relation in leaf phenology, can be differentiated between ring-porous species [29] and those originating from different climatic zones [30].

The important factors limiting the cultivation of plants and introducing alien species to cultivation are frost resistance and the ability to grow in changeable climate [31–34]. Winters in Poland from 16th to 18th centuries were much colder than those in the 20th century, when air temperature measurements become common [35], but also shorter by circa 10 days than in the 1881–1939 period [36]. Phenological studies carried out in Wrocław in 1999–2002 period have shown that the average temperature of winter months was higher compared to year 2020 [37, 38]. The climatic changes were observed in Poland, such asthe increase of average air temperature linked with a longer growing season in autumn. The growing season in the years 1981–2010 (216–220 days) was 3 days longer than in 1971–2000 in Warsaw, Poland [39]. Also, the dates of last spring frost observed at six meteorological stations in Poland were reported statistically significantly earlier from 1.6 to 3.5 days per decade in the years 1961–2020, noted as consecutive days of the year. However, the last spring frost occurred 7–14 days earlier in the years 1991–2020 [40]. However, the lengthening growing season induces more frequent frost days during the growing season (days with minimum temperature < 0 °C) in many areas of the Northern Hemisphere at latitudes greater than 30° N [41]. In the urbanized areas climatic conditions are notably different however, especially the heat island effect is notable, where the average air temperature is higher than in areas surrounding the agglomeration [42]. It is also worth noting that climatic conditions are a more important factor than air pollution regarding induction of phenological changes [43].

*Heptacodium miconioides* was not widely researched so far, despite its status as threatened species. Currently the interest is growing due to the species’ high ornamental value and potential for development of new cultivars. The aim of our research was to identify the phenological phases and the time of their occurrence in *H. miconioides* in temperate zone according to observations carried out at two separate locations and two adverse climatic conditions. We have also assessed the changes in anatomical structure of stems by the end of growing season and during the entry into dormancy period. Our observations will broaden not only the knowledge on *Heptacodium* biology, but also on the subject of entering dormancy period by late flowering plants of temperate climate zone and climate adaptation.

## Material and methods

### Plant material and growth conditions for phenology research

**Fig. 1:**
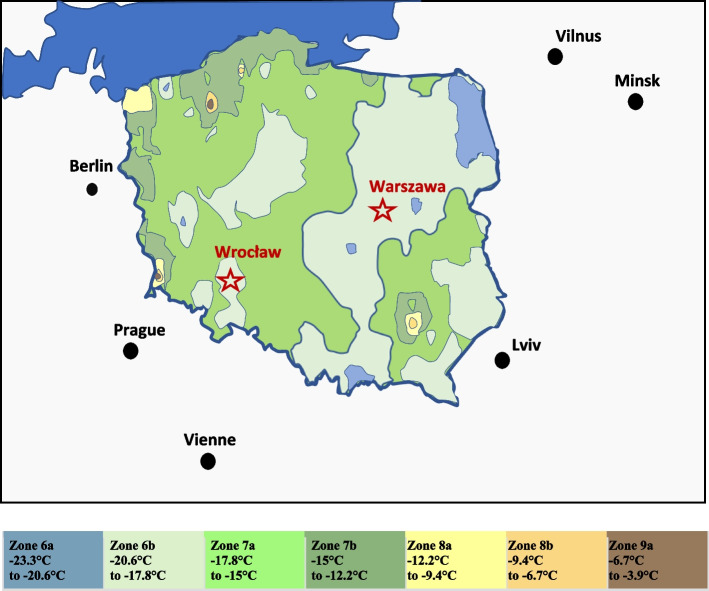
Geographical location of the phenological observation site (Warsaw and Wroclaw), on the background of the zones USDA in Poland [44]

Phenological observations were carried out for two locations (Fig. 1). During the time of the study, as well as during additional field inspections, no frost damage, diseases and pests were observed on the plants. The bushes grew in a sunny and sheltered position, optimal in terms of growing conditions (park layouts). The maintenance procedures were limited to mowing the turf, no pruning, irrigation or fertilization was carried out.

#### Site 1

Two plants of *Heptacodium miconioides* growing in Szczytnicki Park in Wrocław (Poland, 51°N, 17°E) were observed in the years 2012–2013. The age of the specimens can be estimated at approx. 30 years and they grow as a tall shrubs, approx. 4 m. high. The shrubs grew on sandy loam soil, among other low shrubs, in a sunny position, on the north-west side sheltered by a belt of tall trees growing at a distance of approximately 10 m.

#### Site 2

The 5 plants of *Heptacodium miconioides* which were growing in the Polish Academy of Sciences Botanical Garden – Center for Biological Diversity Conservation in Powsin (Poland, 52.6°N, 20.5°E). The age of the specimens can be estimated at approx. 30 years and is characterized by the habit of a tall shrub, approx. 4 m high. The bushes grew on a sandy loam soil, among other low bushes, in a sunny position, on the north-west side sheltered by a belt of tall trees growing at a distance of approximately 10 m. The research was conducted in the period of 2018–2021.

### Climatic conditions

The climatic conditions for the researched sites were as described below.

#### Site 1

Wrocław is located in south-west Poland, at the food of the Sudetes, on the Wrocław Plain, at the very center of the vast Silesian Lowland, in the Wrocław-Magdeburg Glacial Valley which cuts it [45, 46].

**Fig. 2:**
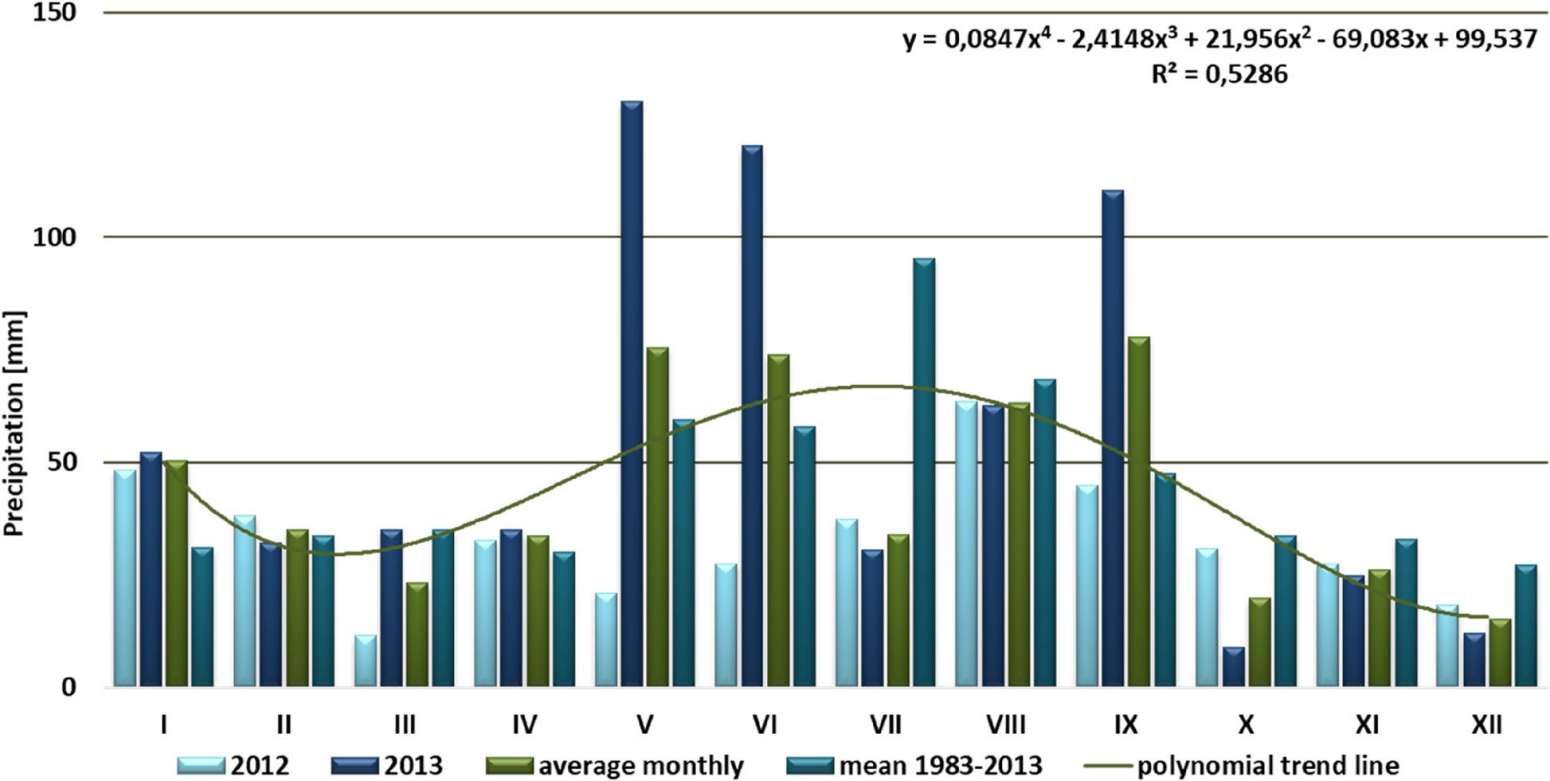
Sum of monthly precipitation in the years 2012–2013, observed at the Wrocław—Airfield meterological station

**Fig. 3:**
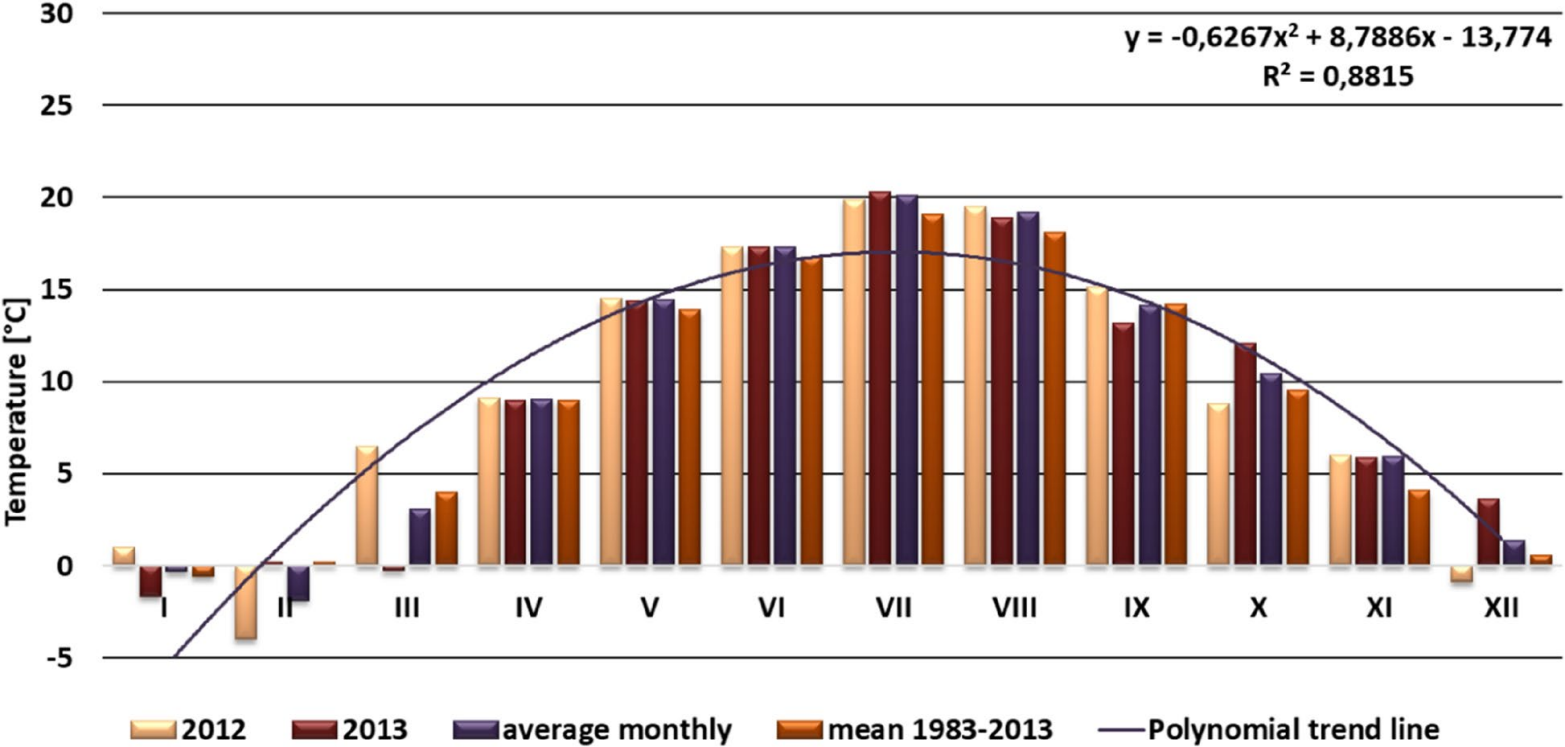
Means of monthly temperatures in the years 2012–2013, observed at the Wrocław—Airfield meterological station

The climate of Wrocław is similar to the transitional zone of temperate latitudes, being subject to both oceanic and continental influences, hence it is characterized by high variability. As Wrocław is located in the south-western part of the country, at the foot of Sudeten, with dominance of winds from south and west, it is among the warmest cities in Poland. There is a distinct spatial variation of climatic and bioclimatic conditions, chiefly temperatures, related to the phenomenon of urban heat island (UHI), understood as an increase in temperature in the city in relation to the surrounding. This phenomenon is typical for large urban-industrial agglomerations. It results from the changes in physical conditions caused by the way of development and use of urban areas, mainly the type and density of buildings, as well as anthropogenic heat energy emission in industrial and municipal processes. Artificial, often hardened surfaces characterized by increased heat capacity and conductivity often prevail in urban areas. Such surfaces easily accumulate heat during the day and then radiate it to their surroundings by night, hence the greatest intensity of UHI effect is observed during cloudless and windless summer nights [45, 47]. The climatic conditions in Wrocław were based on data sourced from Wrocław Airfield metrological station and shown in Figs. 2 and 3.

2012 was remarkably dry in Wrocław, annual precipitation totaled at 401.1 mm; it was therefore 151.1 mm lower than the average for 1983–2013 30 years period. The average precipitation values were lower than long term average for all months except December (Fig. 2). Of those, March was especially dry, with a sum of precipitation of just 11.6 mm. 2013 by contrast was abundant in rainfall, with sum of precipitation at 658.7 mm. It was therefore 106,6 mm more than the average for 1983–2013 30 years period. The driest month was October, with precipitation at 11.9 mm. The precipitation was higher than the long-term average for May and July, though. For the remaining months significant deviation from long term average did not occur (Fig. 2).

2012 was warm in Wrocław, with average yearly temperature at 9.4 °C, so by 0.3 °C higher than the average for 1983–2013 30 years period. The coldest month was February, with average temperature of −4 °C. 2013 was also a warm year, with similar average temperature of 9.4 °C. January was the coldest month, with average of –1.7 °C followed by March at −0.3 °C. By July, August, October, November and December temperatures were higher than long term average (Fig. 3).

Wrocław lies in USDA hardiness zone 6B (minimal average temperatures −20.6 °C–−17.8 °C) [44].

#### Site 2

**Fig. 4:**
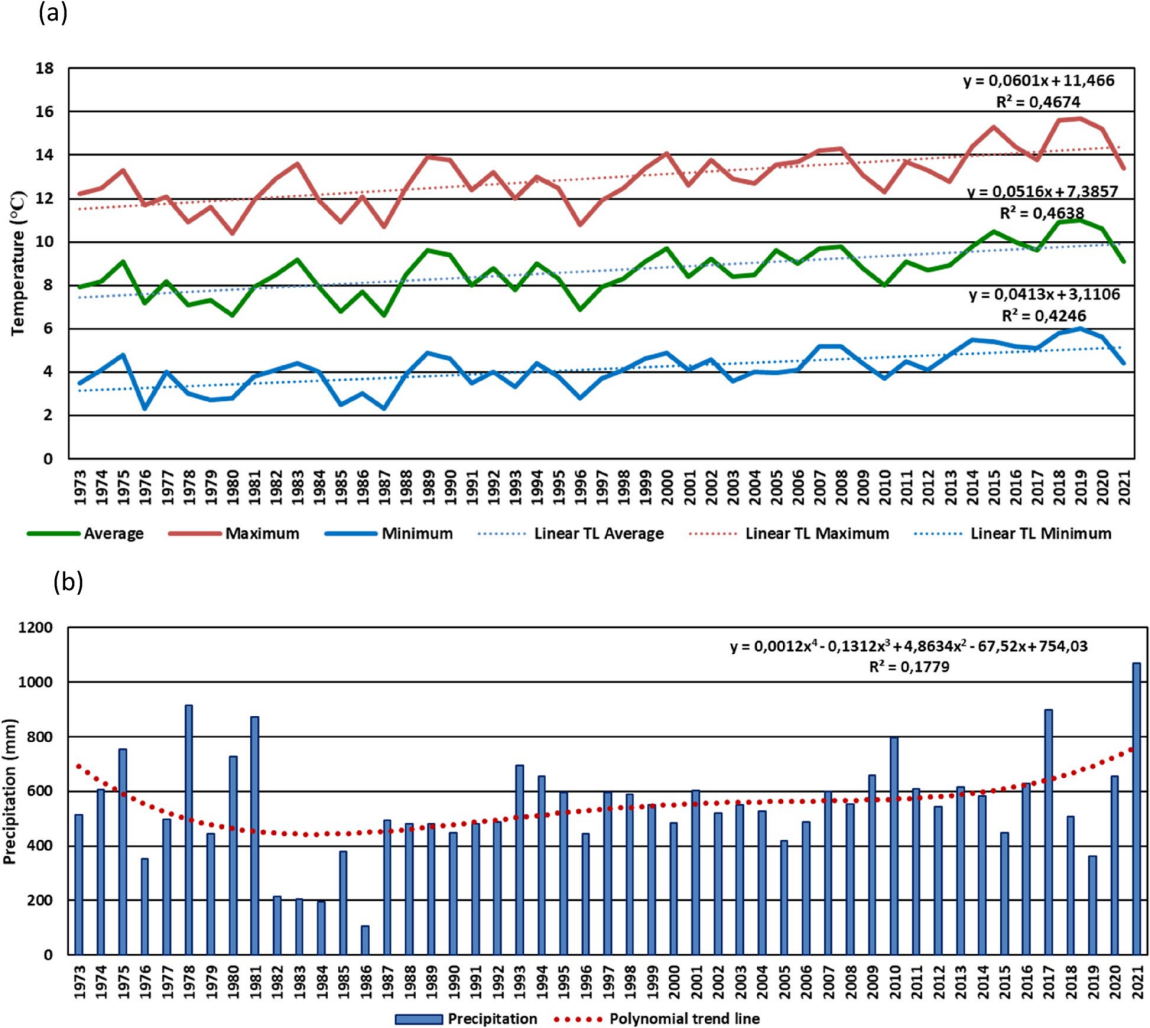
Means of monthly temperatures (average, minimal, maximal) (**a**) and precipitation (**b**) in the years 1973-2021observed at the Warsaw-Okęcie meteorological station

The Polish Academy of Sciences Botanical Garden – Center for Biological Diversity Conservation in Powsin is located by the southern borders of Warsaw (Poland, 52.6°N, 20.5°E) in the Middle Vistula mesoregion [48]. Warsaw, which is a central land region, lies in USDA hardiness zone 6B [44]. The measurements were carried out at the Warsaw-Okęcie meteorological station (52.16°N, 20.96°E, altitude 106 m). This is the closest meteorological station of the Institute of Meteorology and Water Management in Warsaw, 10 km from PAS Botanical Garden in a straight line. The observations so far have shown that in the years 1973–2021 in this region of Warsaw the average monthly temperature increased by approx. 1.5 °C (Fig. 4).

Research carried out in Poland in the 2001–2009 period has proven that the growing season’s length has increased by 8 days on average and by 3 days in Warsaw [39]. Simultaneously, after many years of lasting trend of decreasing yearly precipitation, with significantly lower values for 2018–2020 period, a notable increase in precipitation was observed for 2021 [49] (Fig. 4).

**Fig. 5:**
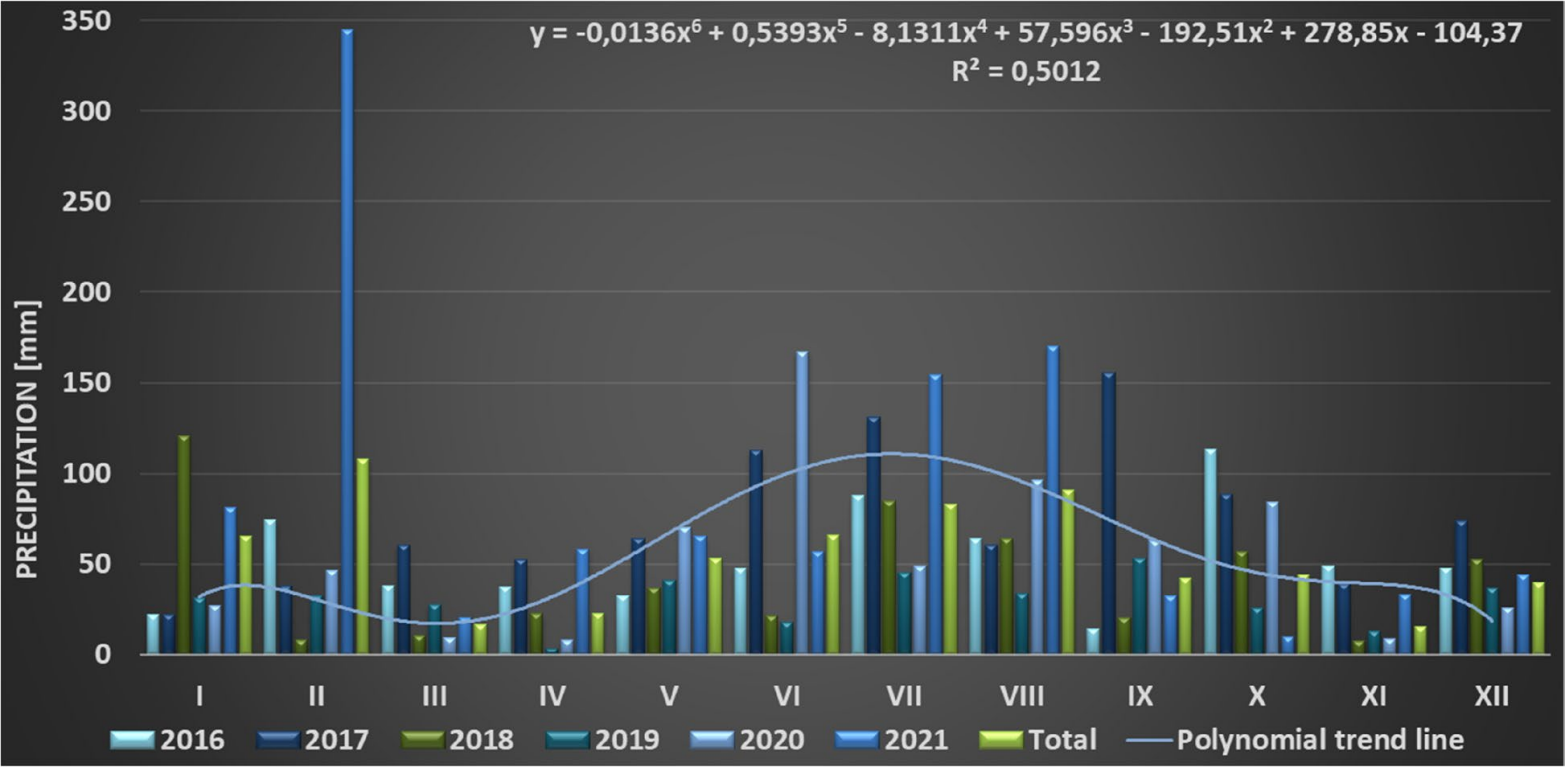
Sum of monthly precipitation in the years 2016–2021, noted at the Warsaw-Okęcie meteorological station

The course of precipitation in the years 2016–2021 in following months is presented in Fig. 5. The polynomial trend line estimation with R-square values of multiple regression were additionally shown. The tendency for an increase in the sum of precipitation was observed in two periods of the year: January and July/August. A disadvantageous drought phenomena were observed in the low sum of precipitation in early spring (March, April) and late autumn (October, November). An extremely high precipitation event was also observed (February 2017) [49] (Fig. 5).

**Fig. 6:**
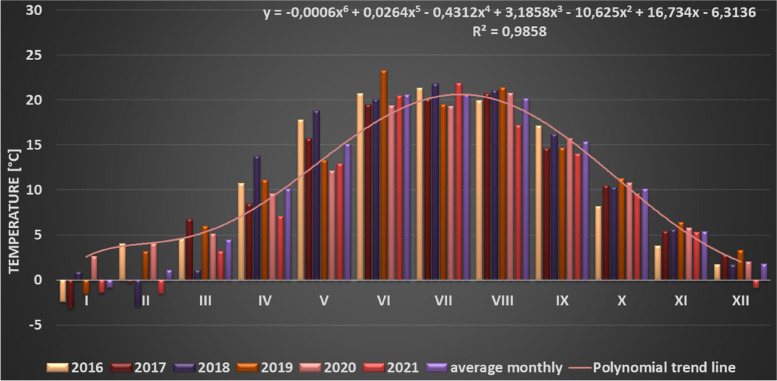
Means of monthly temperatures in the years 2016–2021, noted at the Warsaw-Okęcie meteorological station

During phenological studies carried out in the winter period (December, January, February) the temperature only sporadically dropped slightly below 0 °C (02.2018; 01.2019; 01.2021) (Fig. 6).

### Phenological studies

The *Heptacodium miconioides* was evaluated in the years 2012–2013 in Wrocław (A) and for 2018–2021 in Warsaw in Botanical Garden CBDC in Powsin (B). The chosen phenological stages were recorded according to the basic principles of the BBCH (Biologische Bundesortenamt, CHemische Industrie) scale after winter dormancy or the resting period (BBCH scale 00) from the beginning of bud break to leaf fall and fruit sets according to chosen points of the BBCH scale described for cultivated plants. The phenological development stage in *Heptacodium miconioides* were assigned when at least 50% shoots on the plants it represents reached it [50, 51].

The following principal and secondary growth stages were studied according to the site of observations (respectively A and/or B) and BBCH scale [50]:

- 00 – winter dormancy (B);
- 01 – beginning of bud swelling (B);
- 07 – beginning of bud breaking (B);
- 09 – leaf buds have the green tips (A,B);
- 10 – first leaves separated (A, B);
- 11 – first leaves unfolded (B);
- 12 – 2 true leaves unfolded (A,B);
- 15 – 5 true leaves unfolded (B);
- 21 – first side shoot visible / first tiller visible (B);
- 31 – 1 node detectable (B);
- 32 – 2 nodes detectable (B);
- 33 – 3 nodes detectable (B);
- 51 – inflorescence of flowers visible, beginning of heading (A,B);
- 52 – inflorescence expanded (B);
- 55 – first simple flowers visible: bud flowers (B);
- 56 – first simple flowers visible: bud colorized (B);
- 59 – first flower petals visible (B);
- 60 – first flowers open sporadically (B);
- 61 – beginning of flowering (10% flowers open) (A,B);
- 62 – beginning of flowering (20% flowers open (B)
- 65 – full flowering (50% flowers open) (A,B);
- 67 – the flowers are going to finish blooming (the most of petals fallen/dry) (A,B);
- 69 – the end of blooming (last flowers over blooming, the fruit are visible) (A,B);
- 81 – the first fruit change the color in red (A,B);
- 85 – advanced ripening of fruit coloration (50% fruit change its color in red) (A,B);
- 88 – all the fruits are in red color (A,B);
- 89 – drying up and the fruit abscission (A,B);
- 91 – shoot development completed, the leaves still green (B);
- 92a – beginning of autumn leaf discoloration (change of color from green to yellow in approx. 10% of leaves) (A,B);
- 92b – full autumn leaf discoloration (color change in about 50% of leaves) (A,B);
- 92c – end of full autumn discoloration (color change in approx. 90% of leaves) (A,B);
- 93 – beginning of leaf-fall (A,B);
- 95 – 50% of leaves fallen (A,B);
- 97 – end of leaf fall, plant resting (A,B).

Based on the obtained results of observation of phenological appearances, the calendar of phenological phases of vegetative and generative development was determined [52–54].

### Sample preparation to the anatomy studies

The research material was sourced at the Polish Academy of Sciences Botanical Garden – Center for Biological Diversity Conservation in Powsin.

Research material consisted of stems flowering in the current season (Sa) and 2- to 6-year-old ones (Sb), marked with letter S as above. Collection of stems to the anatomy studies was performed by the last year of observations (2021) at the following periods in 18 days, marked with a letter H, i.e. H1 – 10.09., H2 – 28.09., H3 – 16.10., H4 – 3.11., H5 – 21.11. 10 stems were cut from the east side of each of 5 studied bushes. The annual flowering stems were thin (on average 2–4 mm in diameter), while 2–6-year-old stems were up to 10 mm in diameter. In the case of flowering annual shoots, the sections of the first long internode counted from the base of the shoot development site were secured for research, and in the case of 2–6-year-old shoots—the middle part of the shoot.

The shoot fragments were sectioned with the razor blade, then protected and stabilized in a mixture of anhydrous glycerin and 96% ethyl alcohol (v/v 1:1) until they were used for anatomical analysis at Wroclaw University of Environmental and Life Sciences and Botanical Garden of Wrocław University.

Microscopic slides were prepared from the collected material. Slides were cut with tangential, longitudinal and radial sections into 15–50 μm thick slices with Cryostat (Leica, Buffalo Grove, IL, USA). Moreover, the stems were sectioned for analysis and measuring of the anatomy of their tissues by razor blade.

### Histochemical staining for identification of shoot cells and tissues

The slides were treated by separate methods for screening tissue differentiation and chemical features in cell walls during the process of tissue lignification before the winter dormancy period.

The cross sections of shoots were dehydrated and stained with 0.02% fast green (in 95% ethanol) for 5 min and then without rinsing the slices were treated with acetic acid for 30 s. Then the sections were incubated in 1% safranin O (in 50% ethanol) for 30 min and rinsed with EtOH solution. The slices were dehydrated with 3 changes of 95% and 2 changes of 100% EtOH. The dyes were used to screen chemical differences among and within different cell wall types, where lignified tissue was stained red while unlignified was stained in green or blue [55]. The staining allowed us to detect lignin-rich cell walls, such as middle lamella of tracheids, the secondary wall of compression wood tracheids cellulose rich cell walls (xylem cells, fibers, cambium). The slices were closed in Euparal [56] and were observed under a light microscope.

PAS and toluidine blue staining were used to differentiate the wood, phloem and phelloderm tissues. The slides were dehydrated in a row of increasing ethanol concentrations, then incubated in 0.5% periodic acid solution for 1 h. After rinsing in distilled water, the slides were stained in Schiff dye (Cat. No. 1.09033, Sigma Aldrich) for 1.5 h in darkness. The slices were then rinsed and placed in sodium sulphate solution (5 ml 1N HCL; 5 ml 10% sodium sulphate in H_2_O; 90 ml H_2_O) for 5 min. Then the slices were rinsed again and, stained in toluidine blue solution, rinsed in distilled water and dehydrated in a row of increasing isopropanol concentrations (20%, 40%, 60%, 80%, 100%), then closed in Euparal [56].

The 1.5–3 cm sections of shoots Sa and Sb of all terms H1-H5 were macerated in Franklin solution (30% hydrogen peroxide and glacial acetic acid 1:1; v:v) at 70 °C for 72 h, by the modified method of Gao et al. [57]. Then the slices were washed with distilled water to neutralize them and analyzed under a light microscope. For some of the macerated stems, safranin staining was used, as described above. Slices of stems stained this way were then prepared for observation under the microscope by being closed in a glycerin-gelatin mix. For greater durability, the edges of the coverslip were covered with nitro varnish [58].

### Anatomical evaluation

The studies were carried out using the light Zeiss Primo Star microscope and Zeiss Axioscop (Carl Zeiss AG, Oberkochen, Germany), the analyses were carried out at Department of Horticulture, Wrocław University of Environmental and Life Sciences and Botanical Garden, University of Wrocław. The Slideviewer 2.5 for Windows (3D HISTECH, Hungary) software was used to analyze the sections. The measurements of cells and tissue elements were conducted with the specialist ImageJ (for Windows Version 1.8.0, Microsoft Java, USA) software.

For the stems which flowered in current season (Sa) the width of the xylem, and additionally the vessel diameter, were measured.

For 2 to 6 years old stems (Sb) the width of annual rings was also measured. The differences in woody tissue structure were analyzed for all the annual rings, based on microscopic observations. The identification procedure for analyzed wood was based on the observation of given annual ring along the entire circumference of a stem cross-section. The width of the wood layer, and additionally the early and latewood vessel diameter and vessel area were measured. Additionally, the activity of meristematic tissues—cambium at following intervals H1-H5 was also observed.

The results were referred to wood anatomical features described in the IAWA List of microscopic features for hardwood [59] and bark identification [60].

The surface of vessel cross-section in 1 mm^2^ was calculated for early and late wood in annual wood (Sa), as well as in each ring of perennial shoots (Sb) for five consecutive terms H1-H5. ImageJ (for Windows Version 1.8.0, Microsoft Java, USA) software was used for described measurements.

The number of vessels of metaxylem, secondary wood, early- and late wood were counted in 7–10 field areas of cross sections and converted to the number per 1 mm^2^ [61]. These measurements were conducted for H5 using 10 1-year stems and separately for 6-years old stems.

### Statistical analysis

The phenological observations were general in nature, allowing for the identification of the regularities in the process of vegetative and generative development in the growing season. The results were shown as tables and figures.

The statistical analysis anatomy measurement data were performed using Statistica 13 software package (Statsoft Polska, Cracow, Poland). Analyses of variance (ANOVA) for sets of data groups for anatomical measurements were performed with post hoc Newman-Keuls’s honest significant difference test (α = 0.05). The analyses were carried out for stems of various ages; however the observed ring was always from the same year. A regression analysis of the residual correlation between the width of the annual increment and the sequence of the wood ring on the cross-section and the year of the study was performed. The results of width of annual rings in consecutive years were exposed in one-way analysis of variance with the 95% confidence intervals.

## Results

### Phenology

Described phenological stages were showed in Figs. 7, 8, 9, 10, 11 and 12 and their terms in Figs. 13 and 14. The dates of the above phenological appearances were used to determine the calendar of the following phases in development of *Heptacodium miconioides*:

I. Vegetative (A-C), marked on graphs (Figs. 13 and 14) by triangle, which could be divided into following phases:
  A– Leafing phase (opening of leaf buds—beginning of autumn leaf discoloration): 00, 01, 07, 09, 10, 11, 12, 15, 21, 31, 32, 33, 91; lasting 192–207 days in Wrocław and 206–234 days in Warsaw.
  B– The phase of autumn leaf discoloration (beginning of autumn discoloration—beginning of leaf fall); 92a, 92b, 92c; lasting 15–20 days in Wrocław and 17–24 days in Warsaw.
  C– Leaf fall phase (beginning of leaf fall—end of leaf fall): 93, 95, 97; which ended after 216–221 days in Wrocław and 267–291 days in Warsaw counting from the start of leafing phase.
II. Generative (D-F), marked on graphs (Figs. 13 and 14) by circle, which could be divided into following phases:
  D– Flower bud phase (first flower buds appear—beginning of flowering): 51, 52, 55, 56, 59, 60; which starts at 240–243 day of the year in Wrocław and at 147–161 day of the year in Warsaw.
  E– Flowering phase (beginning of flowering—end of flowering): 61, 62, 65, 67, 69; lasting 29–31 days in Wrocław and 40–68 days in Warsaw.
  F– Fruiting phase (fruit ripening and dispersal): 81, 85, 88, 89; which lasts to 378–384 day of the year in Wrocław and 324–350 day in Warsaw, counting the days from the beginning of previous year.

**Fig. 7:**
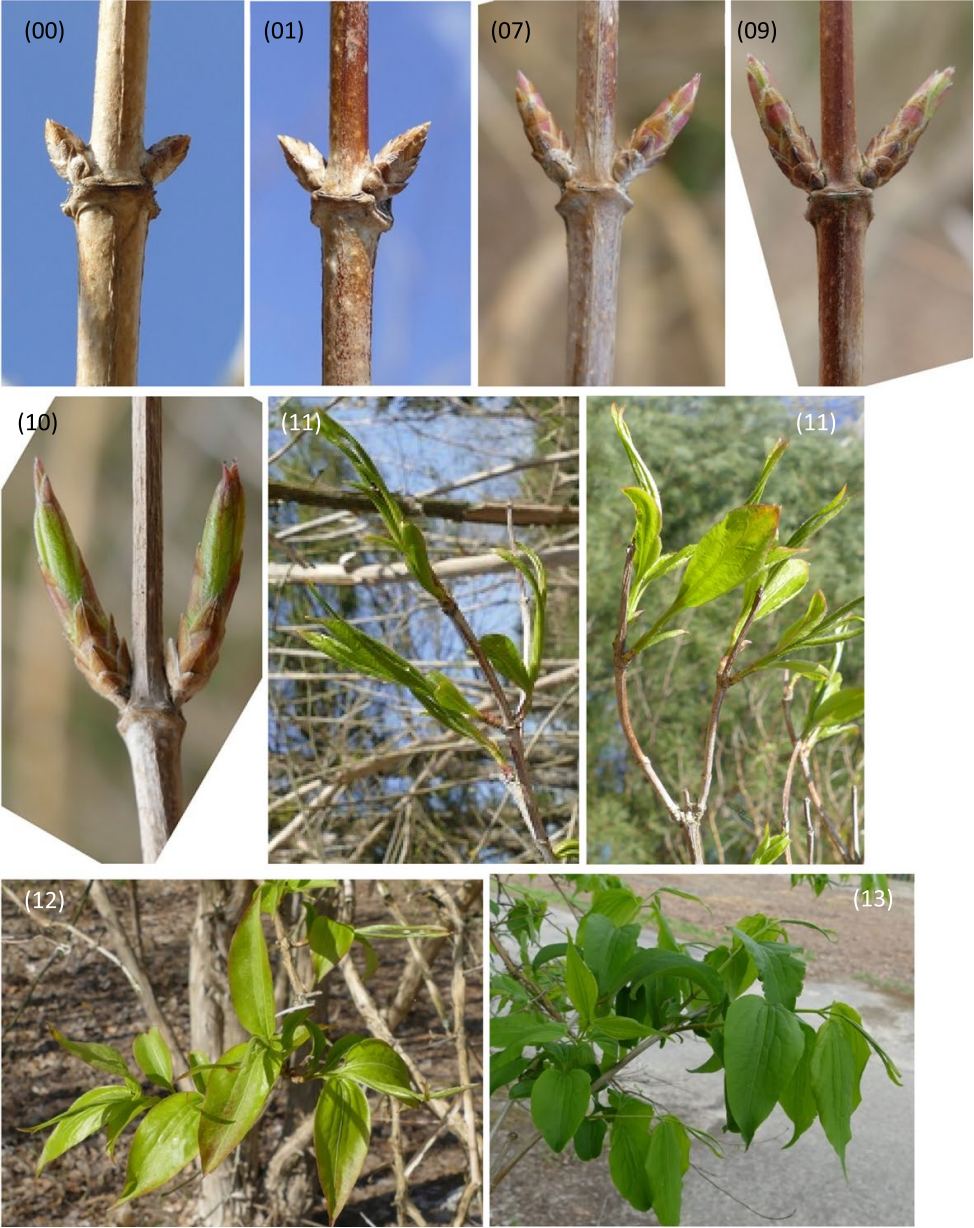
Phenology stages of leaves and shoots development in *Heptacodium myconioides*, according to BBCH scale [50], where: 00 – winter dormancy; 01 – beginning of bud swelling; 07 – beginning of bud breaking; 09 – leaf buds have the green tips; 10 – first leaves separated; 11 – first leaves unfolded; 12 – 2 true pair of leaves unfolded; 13 – 2 true pair of leaves unfolded

**Fig. 8:**
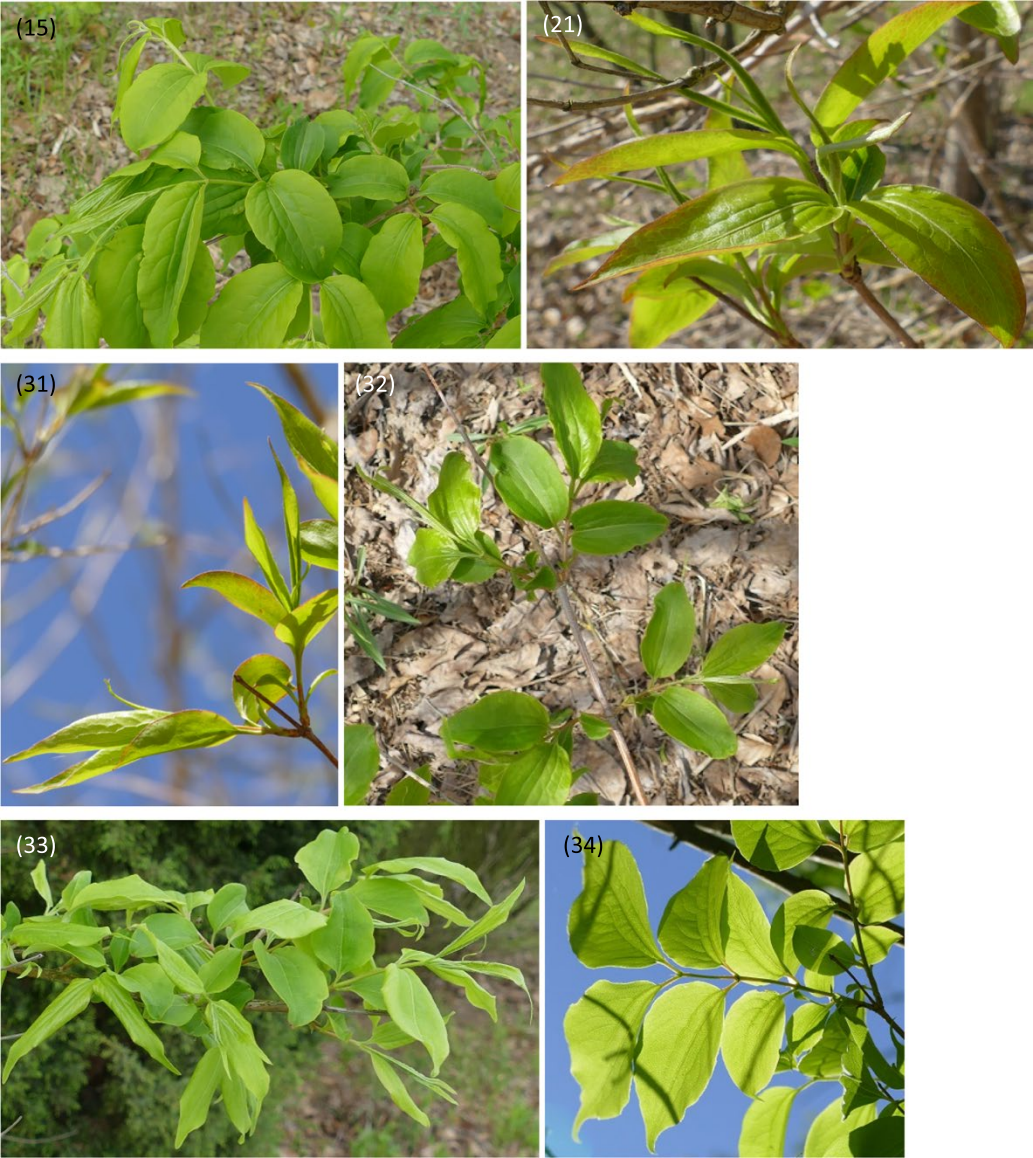
Phenology stages of leaves and shoots development in *Heptacodium myconioides*, according to BBCH scale [50], where: 15 – 5 true pair of leaves unfolded; 21 – first side shoot visible / first tiller visible; 31 – 1 node detectable; 32 – 2 nodes detectable; 33 – 3 nodes detectable; 34 – 4 nodes detectable

**Fig. 9:**
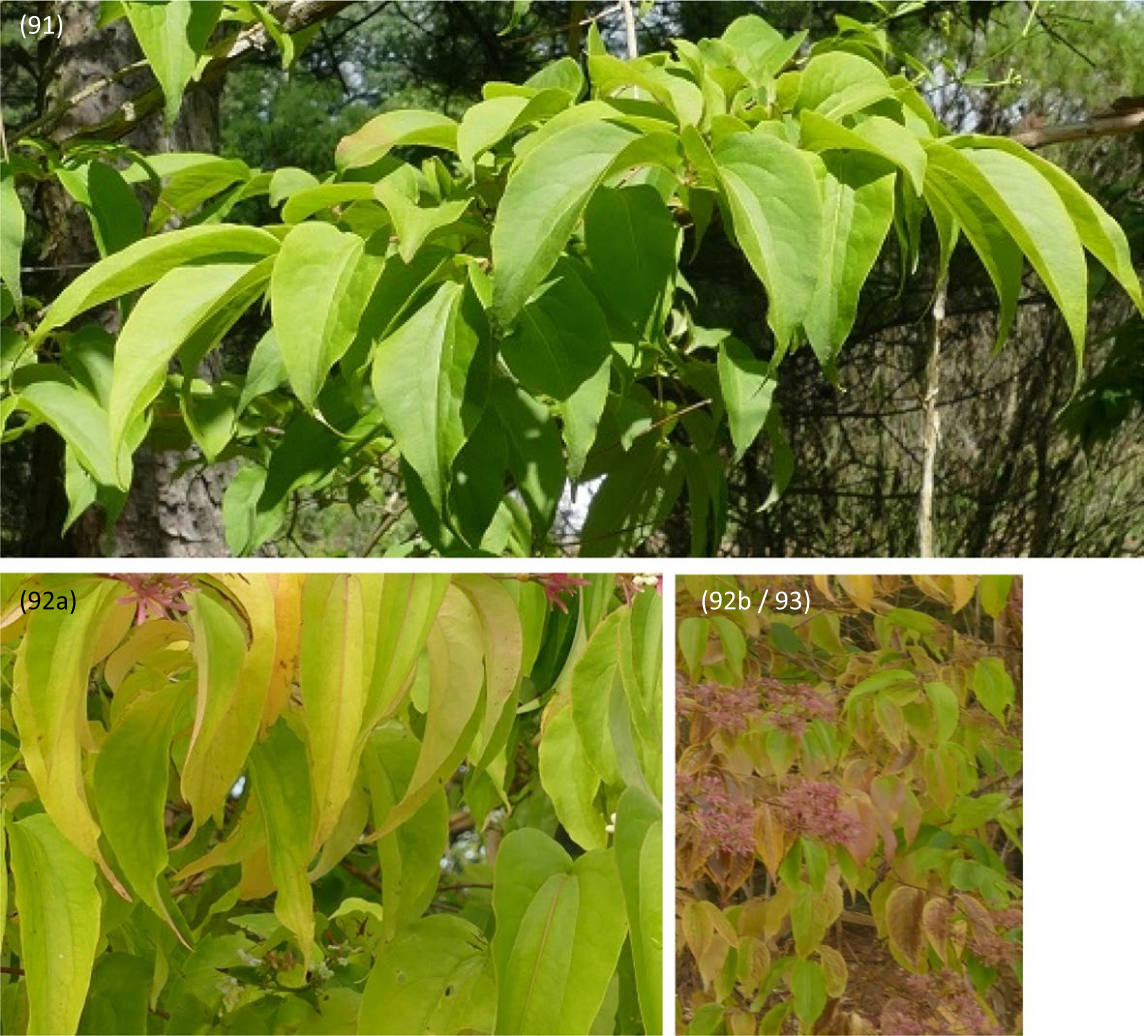
Phenology stages of leaves and shoots development in *Heptacodium myconioides*, according to BBCH scale [50], where: 91 – shoot development completed, the leaves still green; 92a – leaves begin to discolour (change green to yellow colour in 10% of leaves); 92b – leaves discoloration (the colour changed in 50% of leaves); 93 – beginning of leaf fall

**Fig. 10:**
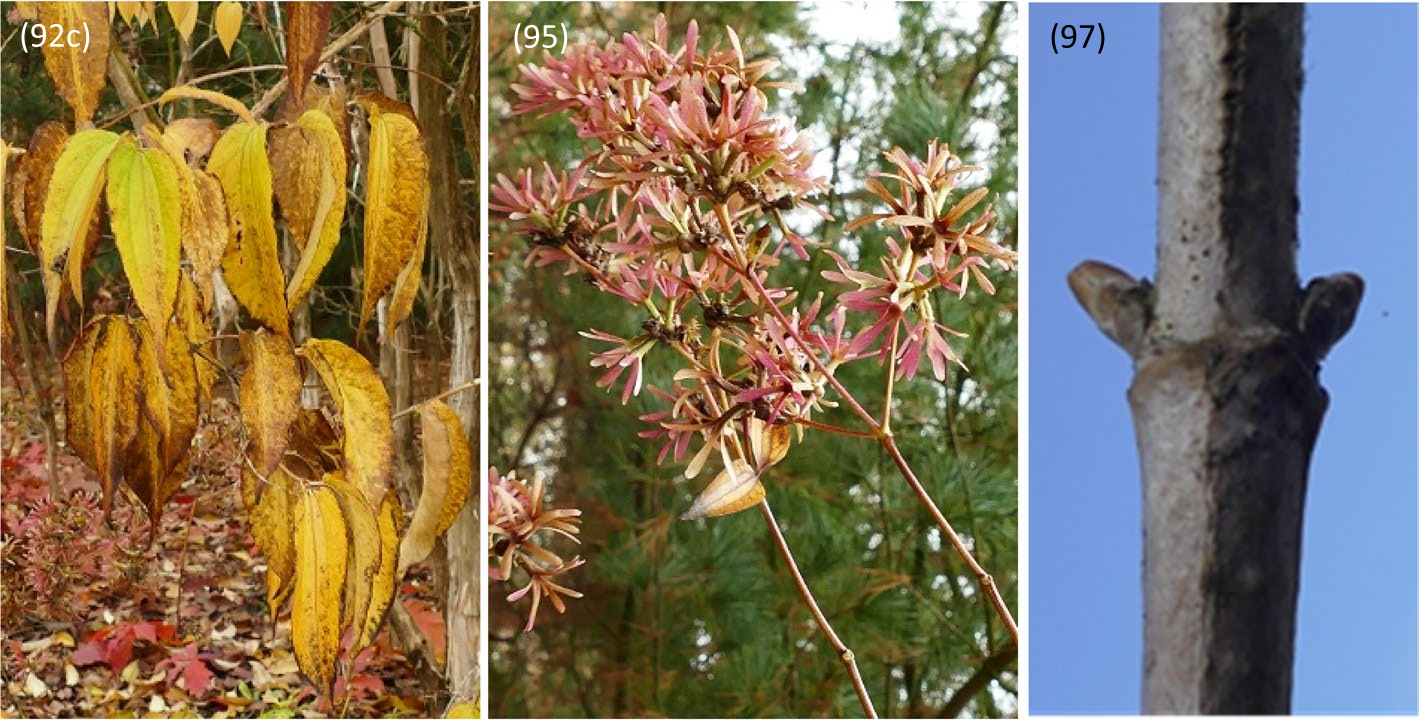
Phenology stages of leaves and shoots development in *Heptacodium myconioides*, according to BBCH scale [50], where: 92c – the end of leaves discoloration (the colour changed in 90% of leaves); 95 – 50% of leaves fallen; 97 – end of leaf fall, plant resting

**Fig. 11:**
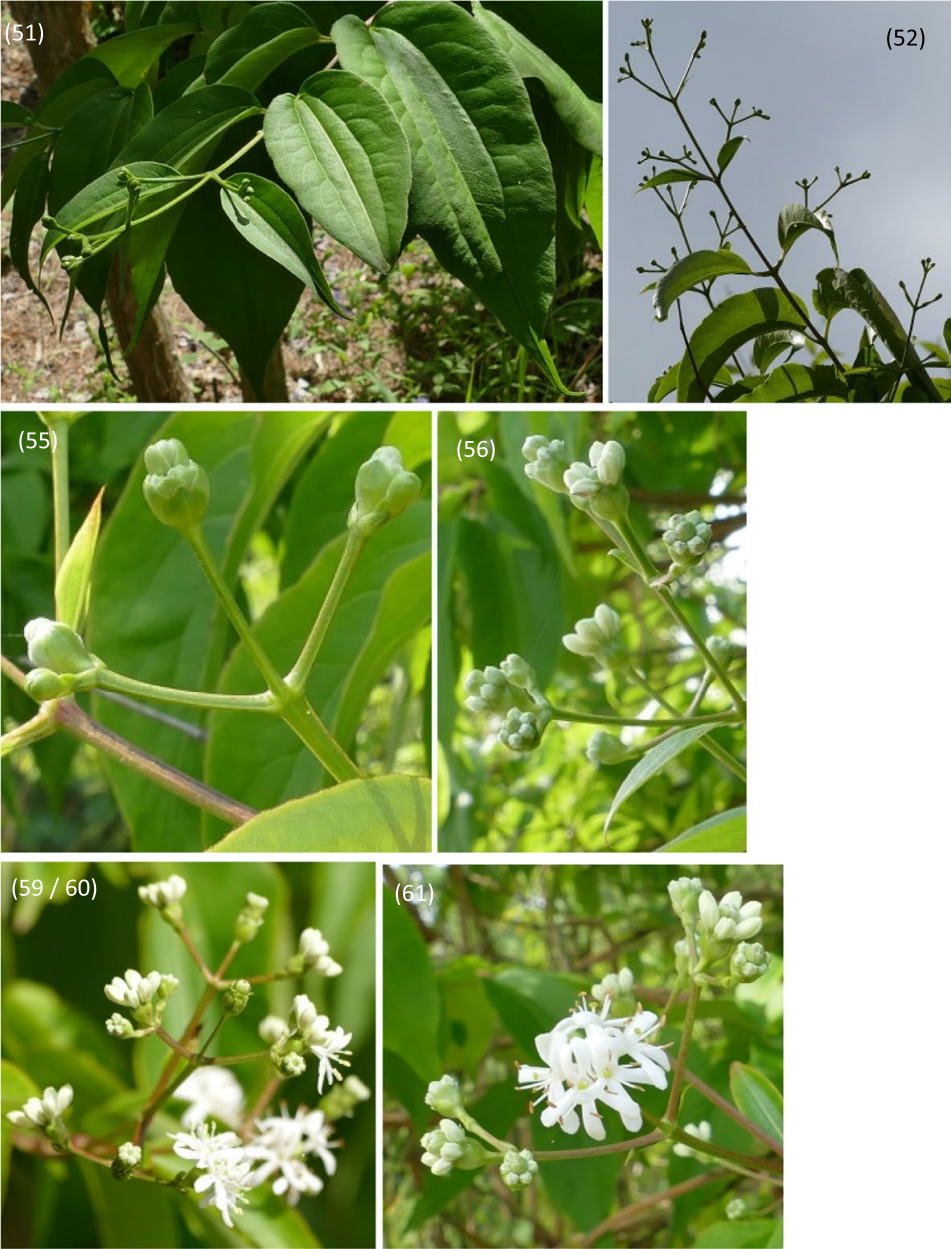
Phenology stages of flowers and fruit development in *Heptacodium myconioides*, according to BBCH scale [50], where: 51 – inflorescence of flowers visible, beginning of heading; 52 – inflorescence developed; 55 – first simple flowers visible: bud flowers; 56 – first simple flowers visible: bud colorized; 59 – first flower petals visible; 60 – first flowers open sporadically; 61 – beginning of flowering (10% flowers open)

**Fig. 12:**
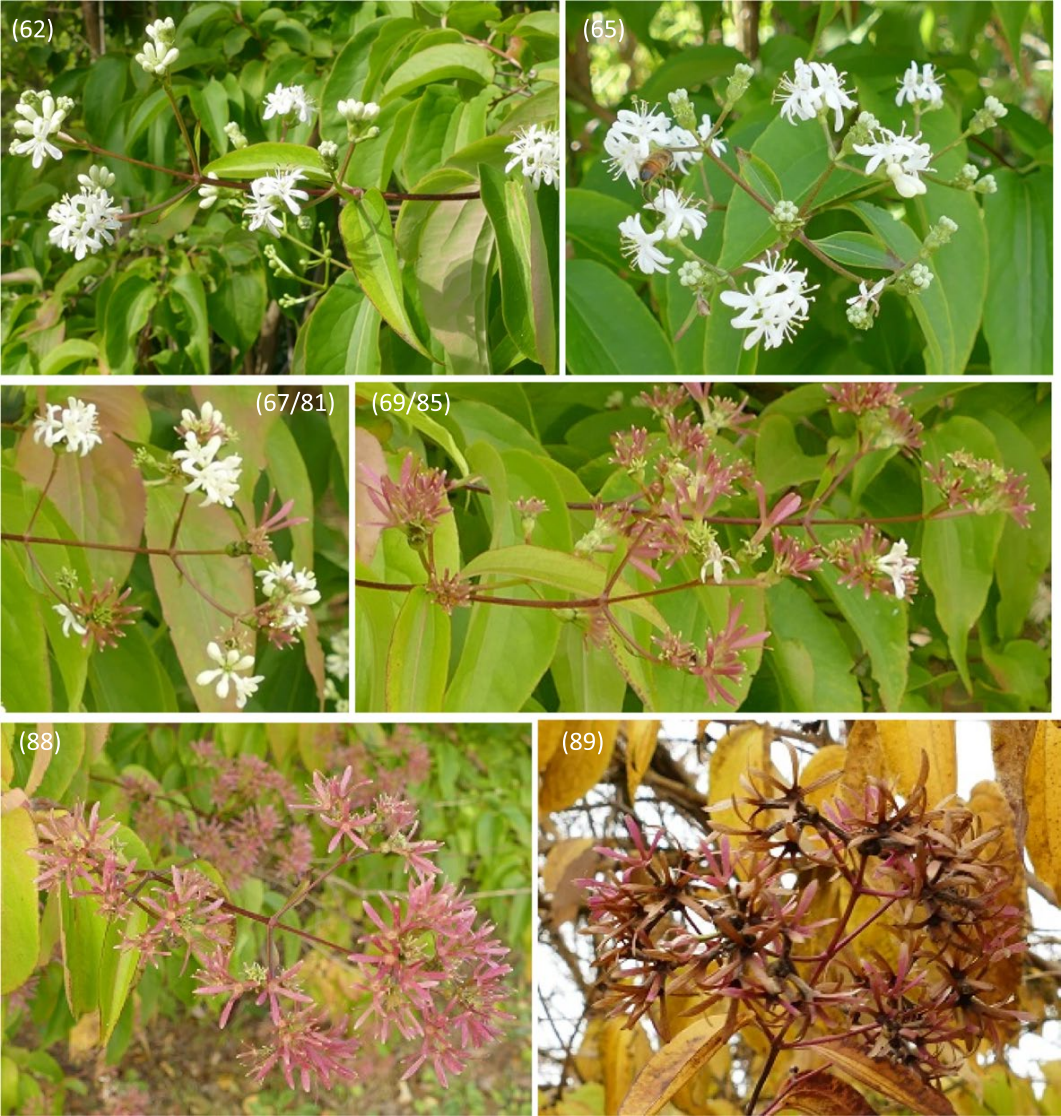
Phenology stages of flowers and fruit development in *Heptacodium myconioides*, according to BBCH scale [50], where: 62 – beginning of flowering (20% flowers open; 65 – full flowering (50% flowers open); 67 – the flowers are going to finish blooming (the most of petals fallen/dry); 69 – the end of blooming (last flowers overblooming, the fruit are visible); 81 – the first fruit change the colour in red; 85 – advanced ripening of fruit coloration (50% fruit change its colour in red); 88 – all the fruits are in red colour; 89 – drying up and the fruit abscission

**Fig. 13:**
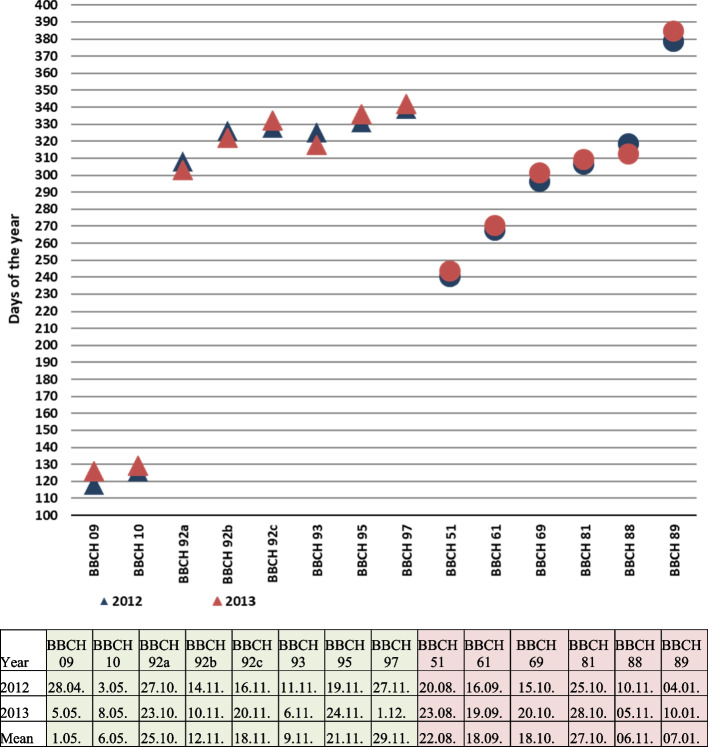
Phenology stages in *Heptacodium myconioides* in Wrocław localization, according to BBCH scale [50], modified and described in manuscript. The number of the days of the Fig are the number of the days in the year (1–31 January; 32–59 February; 60–90 March; 91–120; April; 121–151 May; 152–181 June; 182–212 July; 213–243 August; 244–273 – September; 274–304 October; 305–334 November; 335–365 December; 366–396 January next year). ∆ – stages of leaves and shoots development; ◯ – stages of flowers and fruits development

**Fig. 14:**
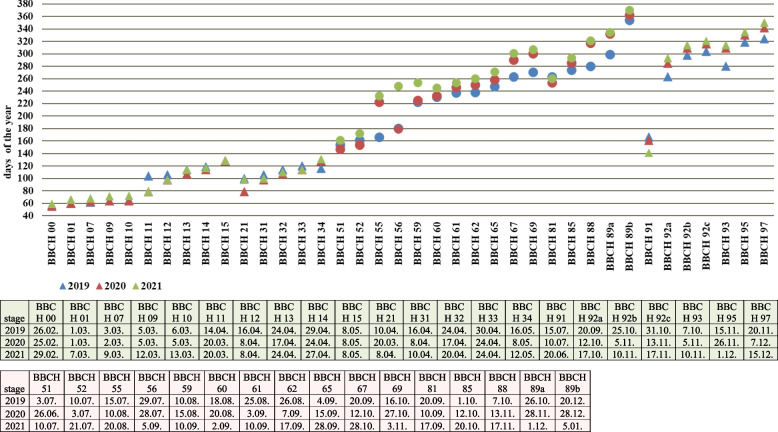
Phenology stages in *Heptacodium myconioides* in Warsaw localization, according to BBCH scale [50], modified and described in manuscript. The number of the days of the Fig are the number of the days in the year (1–31 January; 32–59 February; 60–90 March; 91–120; April; 121–151 May; 152–181 June; 182–212 July; 213–243 August; 244–273 – September; 274–304 October; 305–334 November; 335–365 December; 366–396 January next year). ∆ – stages of leaves and shoots development; ◯ – stages of flowers and fruits development

In the period of 2012–2013 delayed start of vegetation in Wrocław was caused by lower average temperatures in February, contrary to those in Warsaw. However, higher temperature of March in Wrocław in 2012 did not cause an earlier development of leaf buds. It is highly likely that earlier development of leaf buds by the bushes in Warsaw lead to earlier formation of inflorescences, however the actual flowering took place at a comparable date at both sites. In Warsaw the flowering phase lasted a few days longer, albeit the fruit ripening and dispersal phase ended earlier than in Wrocław (Figs. 13 and 14).

### The one-year stem anatomy (Sa)

**Fig. 15:**
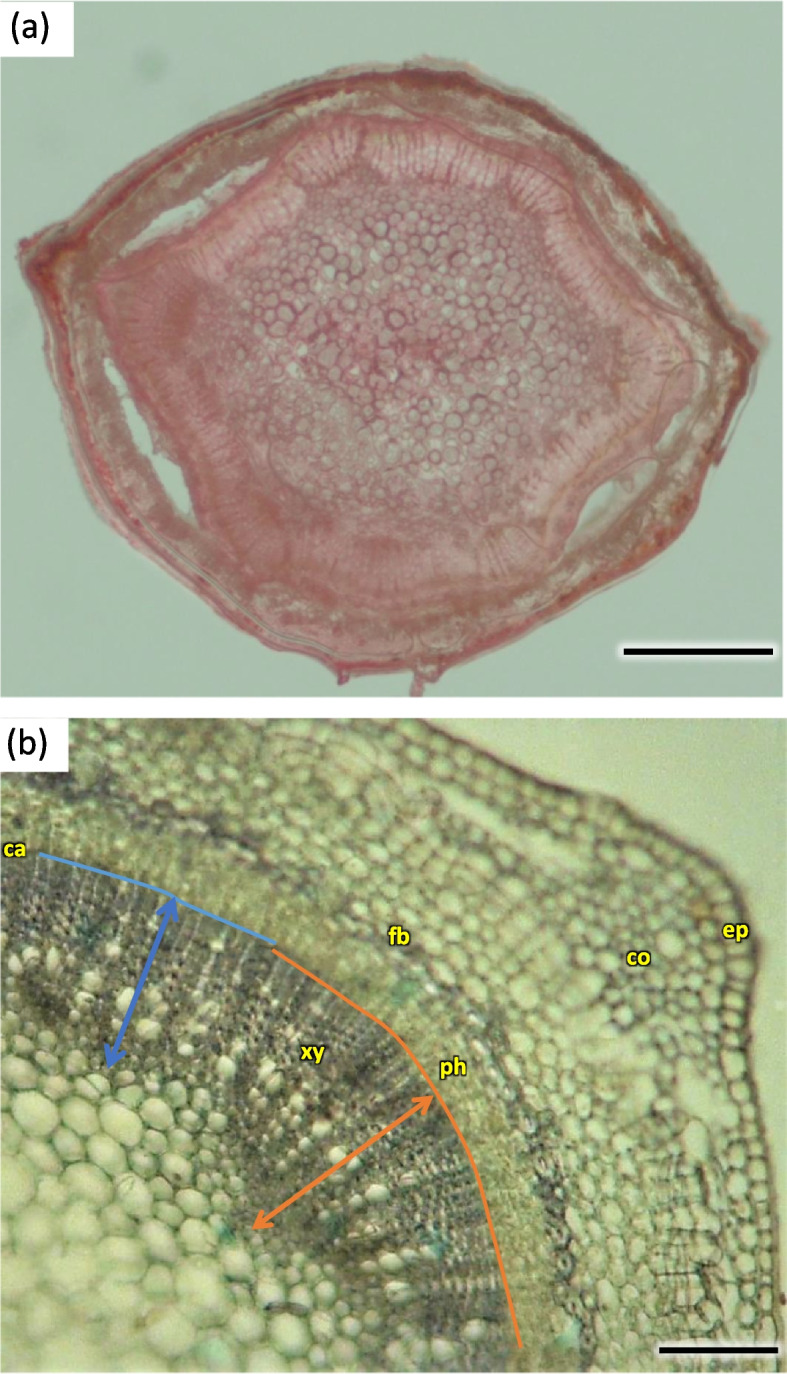
Transverse sections of the 1-year stem of *Heptacodium myconioides*: **a** asymmetrical flattened shoot, scale bar 500 µm; **b** detail of areas with visible asymmetry (orange line – vascular bundle in the asymmetry place; blue line– vascular bundle in the place beyond asymmetry place), H4, scale bar 200 µm. Designations: ca – cambium; co – cortex; en – endodermis; fb – band of phloem fiber; ph – phloem; xy – xylem

Annual flowering stems H1 were slightly flattened on the radial section, with axial symmetry, with characteristic bulges resulting from the uneven growth of the conductive bundles and the growth of parenchymatic tissue cortex. Conductive bundles with a characteristic regular fit to the asymmetrical shape were visible on the cross-section of the shoot, but the bundles themselves of unequal width and length. On a radial section in the circumference, the bundles were arranged in a closed ring. The ring of wood was 1/3 of the width of the radial diameter of the shoot (Fig. 15).

**Fig. 16:**
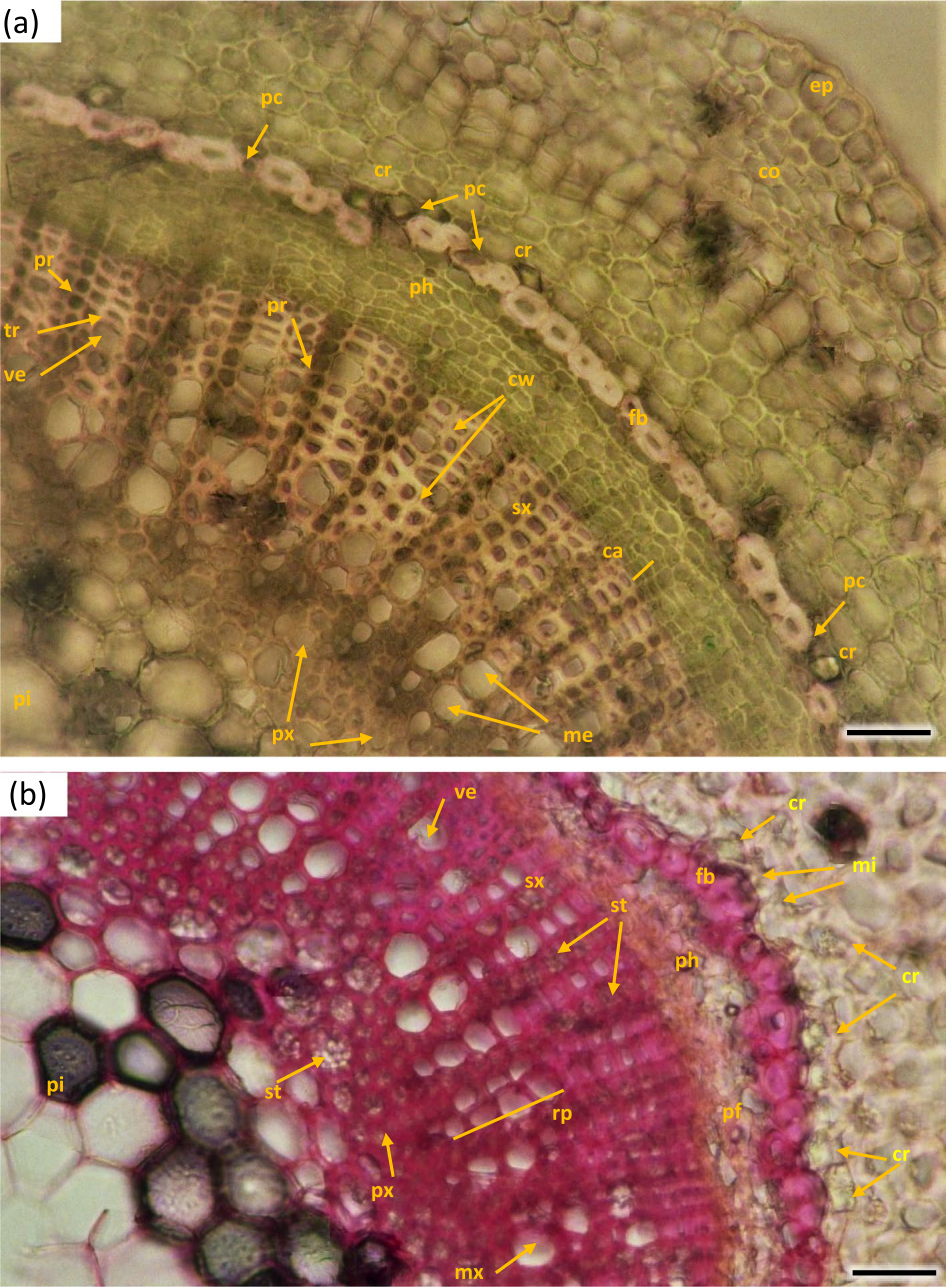
Transverse sections of *Heptacodium myconioides* 1-year stem: vascular bundle with visible metaxylem H1 (**a**) and H5 (**b**). Designations: ca – cambium; co – cortex; cr – crystals, druids; cw – thin and thick wood tissue cell walls; ep – epidermis; fb – band of phloem fibers; mx – metaxylem vessel;; mi – mineral inclusions; pc – passage cell; pi – pith; pf – phloem fibers; ph – phloem; pr – pith ray; px – protoxylem; rp – radial pattern; sx – secondary xylem; st – starch; tr – tracheids; ve – secondary xylem vessel. Scale bars 100 µm

**Fig. 17:**
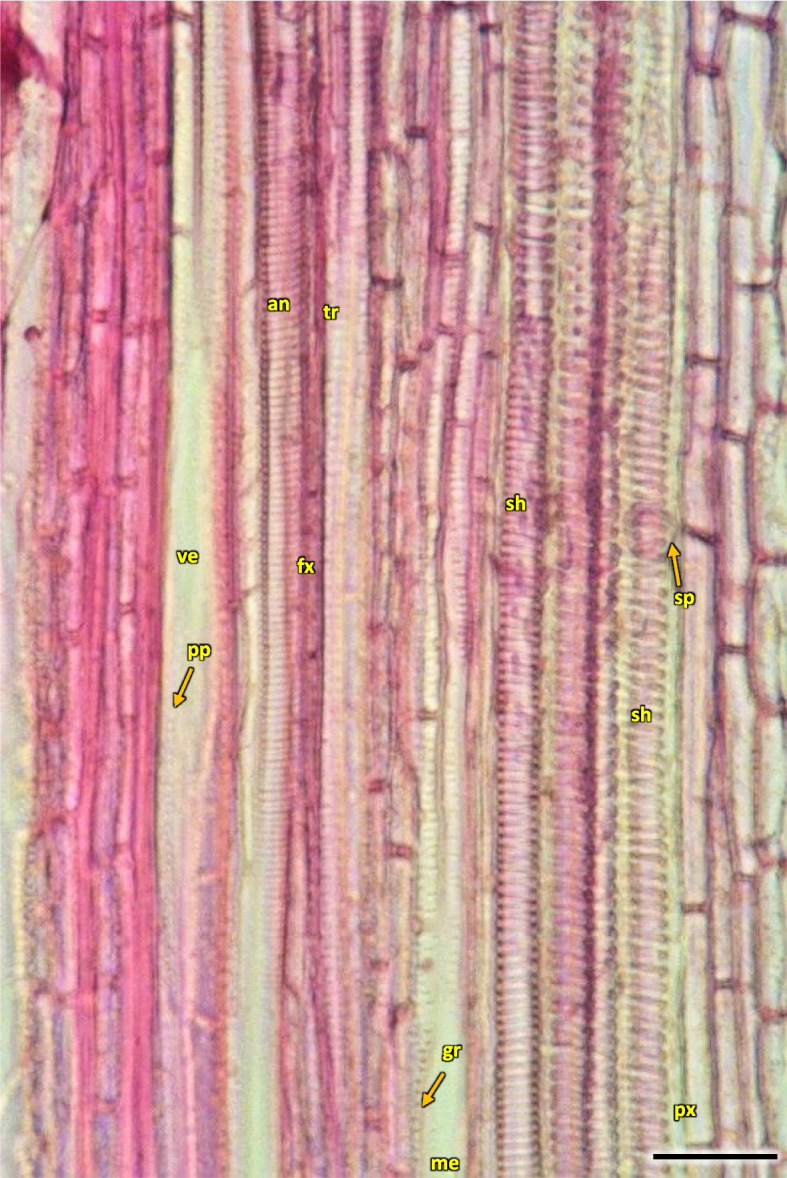
Longitudinal radial section of the 1-year stem H1 in *Heptacodium myconioides*. Designations: an – annular thickening of metaxylem; gr – gradual vessel tail, scalariform thickening and plate (arrow); me—metaxylem vessel; pp – simple pitted vessel; px – protoxylem; sc – scalariform thickening of metaxylem vessel; sh – helical / spiral thickening of protoxylem vessel; sp – simple plates (arrow); tr – tracheids; ve – secondary xylem vessel. Scale bar 100 µm

The width of metaxylem layer was on average 325.2 µm, and the number of vessels per 1 mm^2^—909.2, with average surface of 2478.1 µm^2^ (Table 1). Metaxylem vessels were usually arranged singularly in radial pattern by 4–7, sometimes turning into two, rarely three rows. Each group contained 5–12 rows of vessels arranged in parallel. The farther from the axis of the stem, the larger the diameter of the vessels was (Fig. 16). On average metaxylem vessels (Sa) were 63.1 µm in diameter (Table 1) and 300–500 µm long. The vessels within one row had significantly greater diameter the further they were from the center of the steam (Fig. 16). The outline of solitary vessel was rounded. Metaxylem was significantly lignified, evidenced by pink-red color visible in staining performed for all the terms of stem collection H1-H5. On the longitudinal section vessel walls were usually spiral or annular. Vascular cells connected straight or less frequently with spiral thickening of protoxylem vessels and scalariform perforation plates in metaxylem (Fig. 17).

**Table 1:** Wood characters of the 1-year stems of *Heptacodium miconioides* harvested in 2021 year

**Layer**	**Average of the number of vessels in 1 mm**^**2**^	**Average of vessel area (µm**^**2**^**)**	**Average of vessels diameter (µm)**	**Width of layer (µm)**
Metaxylem	909.2^1^ a ± 15.6	2478.1 b ± 18.7	63.1 a ± 9.4	325.2
Secondary xylem	234.3 b ± 15.8	2569.0 a ± 19.0	45.9 b ± 9.3	224.8

Different letters indicate significant differences between metaxylem and secondary xylem features. The Newman-Keuls’s honest significant difference test (α = 0.05) was used

**Fig. 18:**
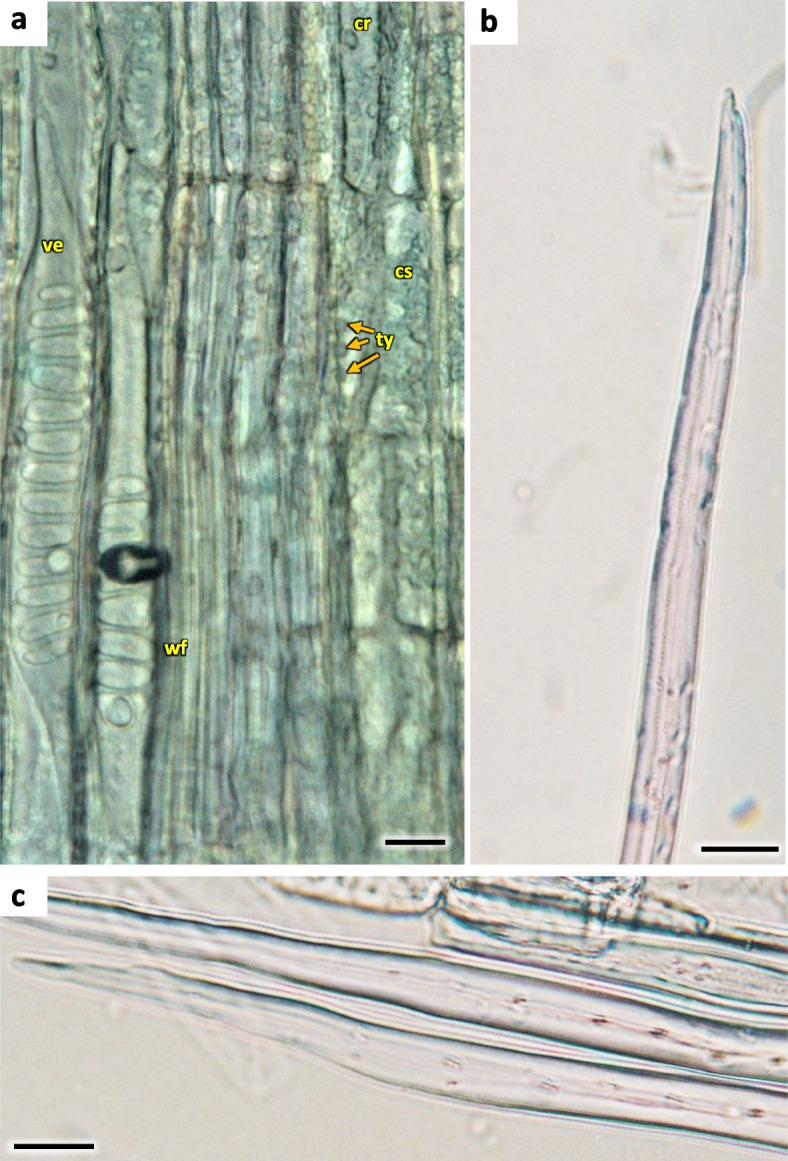
The 1-year stem H1 in *Heptacodium myconioides*, **a** the metaxylem vessel with scalariform thickening (longitudinal radial section); the part of tracheid (after maceration) of **b** protoxylem and **c** metaxylem. Designations: cr – crystals; cs – crystal sand; ty – tylosis; ve – vessel; wf – wood fibers. Scale bars 100 µm

The width of secondary xylem was on average 224.8 µm (Table 1) and it constituted about half of the section of wood tissue on the section of wood harvested at stages H1-H5 in the year 2021 (Fig. 15). The average number of vessels per 1 mm^2^ was 234.3 (Table 1). Angular vessels were partly solitary, partly in radial multiples of 2–4, or very small clusters. Vessels were angular, simple pitted or scalariform, irregular in shape, with average diameter of 45.9 µm (Figs. 17 and 18; Table 2). Tracheids of the secondary wood were angular, irregular in shape, with small internal cross-section area, tightly arranged (Fig. 17). Among them, single vessels were arranged around the circumference in a fairly regular band, with diameter of 45.94 µm (Table 1). The last off-axis rows of wood cells were slightly thinner and slightly flattened, which indicates that the lignification process was still incomplete. The lignification process was progressing over time and only in the H5 term can it be considered fully completed with thick cell walls of tracheids, wood fibers and vessels (Figs. 16 and 18).

**Table 2:** Wood characters in the 6-years stems of *Heptacodium miconioides* harvested in 2021 year

**Trait**	**Average of the number vessels / 1 mm**^**2**^	**Average of vessels area (µm**^**2**^**)**	**Average of vessels diameter (µm)**	**Width of wood layer (µm)**
**1 ring (2016)**				
Metaxylem	274.8 c ± 5.3	991.4 a ± 9.8	33.1 bcd ± 1.4	116.9 c ± 7.6
Secondary xylem	234.7 d ± 4.4	450.3 d ± 16.6	25.9 def ± 1.8	330.8 a ± 28.1
**2 ring (2017)**				
Early wood	342.8 b ± 6.3	465.7 d ± 10.6	38.0 abc ± 1.9	121.2 c ± 2.8
Late wood	236.0 d ± 9.6	420.8 de ± 8.6	26.5 def ± 1.3	323.7 a ± 11.2
**3 ring (2018)**				
Early wood	285.8 c ± 2.7	682.1 b ± 8.4	44.1 a ± 1.8	77.1 c ± 2.5
Late wood	157.8 gh ± 3.4	418.8 de ± 7.7	34.6 bcd ± 1.8	204.8 b ± 4.8
**4 ring (2019)**				
Early wood	178.3 f ± 2.9	449.3 d ± 5.1	43.9 a ± 3.7	103.6 c ± 6.6
Late wood	144.8 h ± 5.4	378.7 e ± 8.2	39.9 ab ± 3.1	184.1 b ± 3.7
**5 ring (2020)**				
Early wood	272.7 c ± 2.7	672.4 b ± 10.5	32.3 bcd ± 1.5	63.1 c ± 4.6
Late wood	166.8 fg ± 5.2	276.6 f ± 7.4	19.6 f ± 2.6	98.5 c ± 11.0
**6 ring (2021)**				
Early wood	361.6 a ± 7.4	510.0 c ± 6.6	29.4 cde ± 1.5	109.8 c ± 8.3
Late wood	216.8 e ± 3.2	233.0 g ± 11.6	21.9 ef ± 2.4	64.4 c ± 3.5

Different letters indicate significant differences between early and late wood features. The Newman-Keuls’s test (α = 0.05) was used

**Fig. 19:**
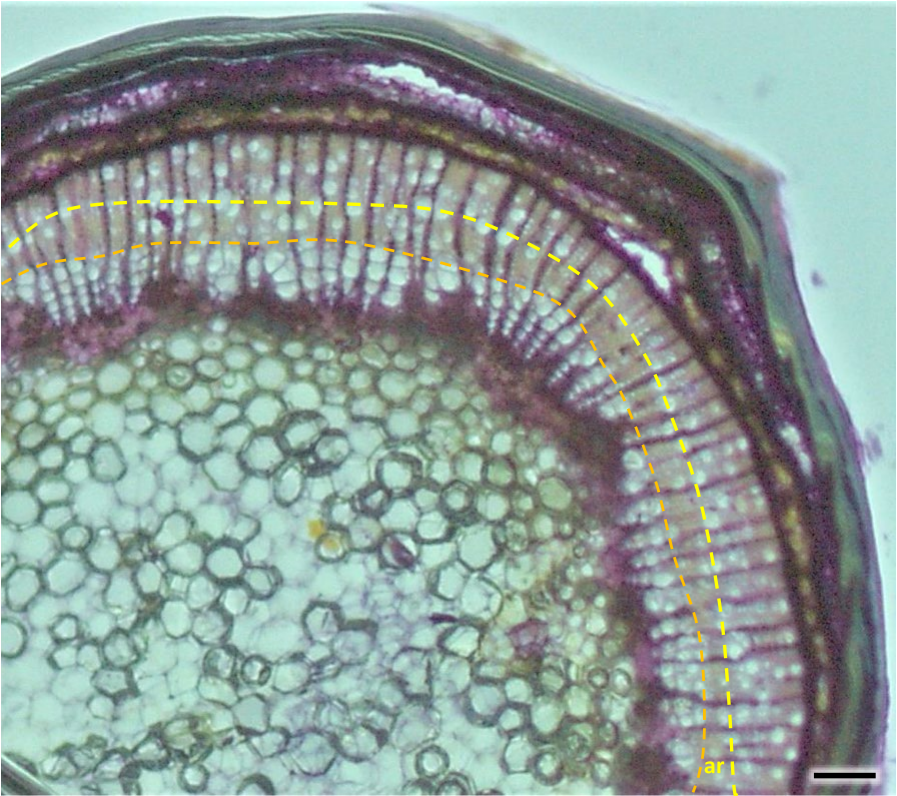
The radial section of the 1-year stem H4 in *Heptacodium myconioides* with the visible band of vessels. Designations: orange line – the border of the metaxylem; lemon line – the additional rows of vessels in secondary xylem; ar – the area between lines with single vessels. Scale bar 200 µm

For some of the radial cross-sections of annual wood in H1-H5 stage one more band laying beyond the sphere of metaxylem was visible, in which more vessels were produced by the cambium. This band was visible as a row of cells on the circumference (Fig. 19). The regular, arranged in series of 5–6 vessels of early wood divided by pith rays were visible in the cross-section of those stems, then a band with thick-walled tracheids dominating the structure, and after it a band of densely packed vessels, finally a band of thick-walled tracheids and sparse vessels (Fig. 16b).

**Fig. 20:**
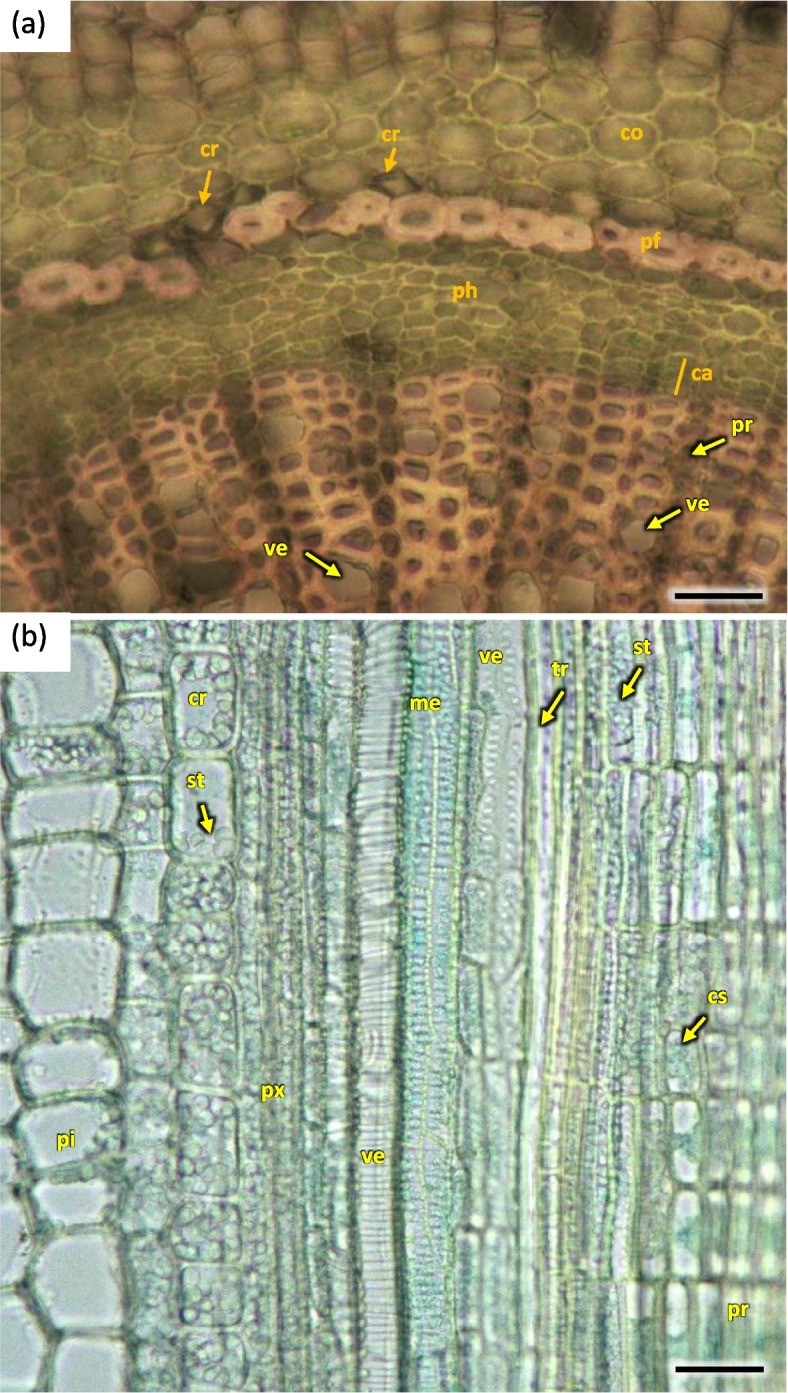
Transverse (**a**) and longitudinal radial (**b**) sections with cambium and phloem layers in the 1-year stem H1 of *Heptacodium myconioides*. Designations: ca – cambium; cr – crystals; cs – crystal sand; me – metaxylem vessel; pf – band of phloem fibers; ph – phloem; pi – pith; pr – pith rays; px – protoxylem; st – starch grains; tr – tracheids; ve – xylem vessel. Scale bars 100 µm

Pith takes 1/2 to 2/3 of the diameter of radial cross-section of stem at H1 term. It consists of large cells, irregular in size and shape, greatest at the center and diminishing in diameter when approaching early wood ground tissue and endoderm. The ground tissue is formed from cells rich in starch (Figs. 15 and 16). Parallel rows of 2–3 vessels are divided by 1 row medullary rays. From the place of formation of first bundles and vessels two or three rows wide pith rays are visible, running continuously from pith ground tissue to phloem. Many granules of starch were visible in piths (Figs. 15, 16 and 20).

During the H1 stage cambium on the circumference of stem was forming a layer of flat cells, without any traits indicating fast divisions and differentiation of wood and phloem tissues cells on the entire circumference. However, in some places sites of active cambium were visible, especially in asymmetric structures. In the radial cross-section storied structure of cambium layer was clearly visible (Fig. 16a).

Prismatic, star shaped crystals were visible in some of the pith rays, probably of calcium oxalate (Figs. 16 and 20).

**Fig. 21:**
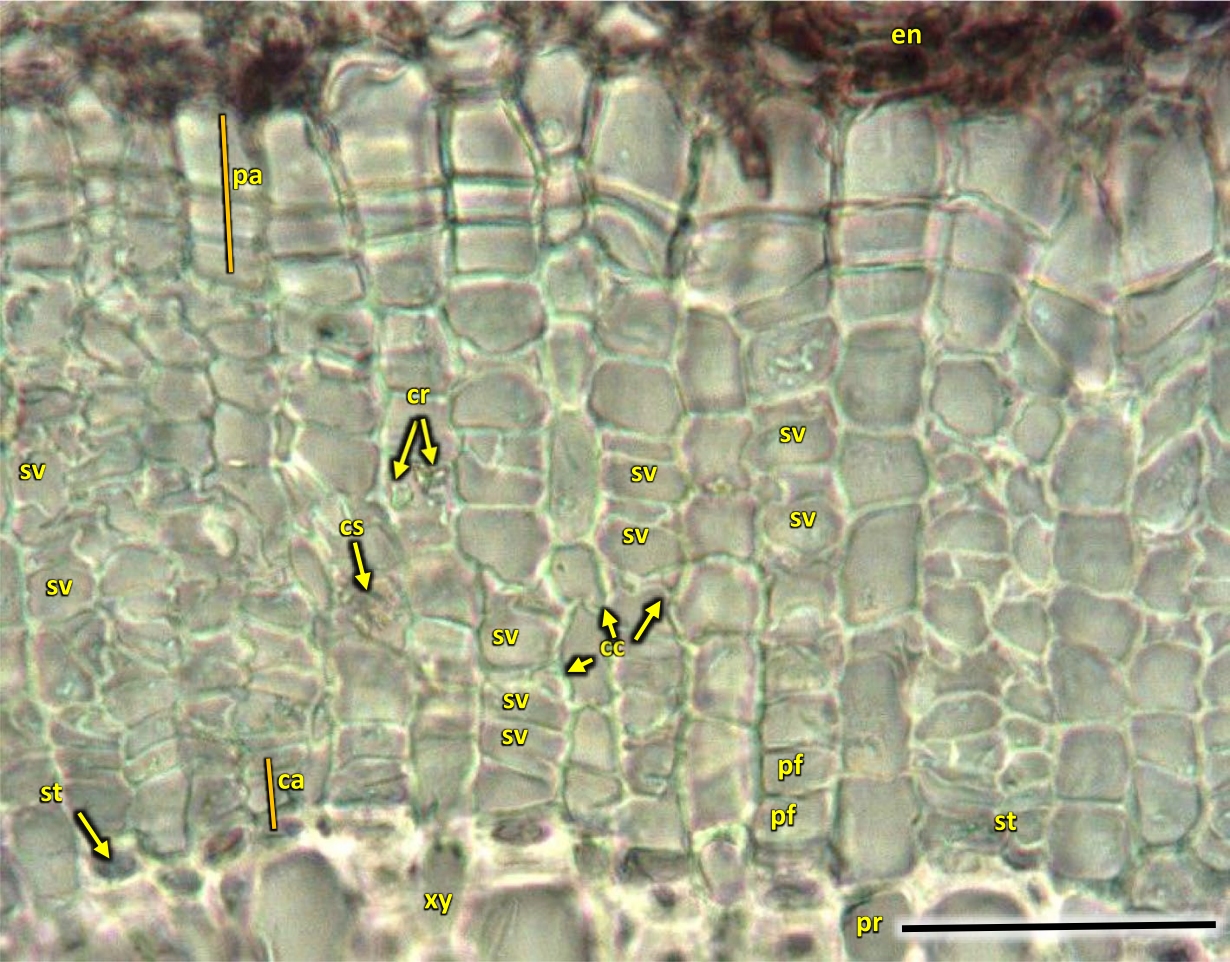
Transverse radial section with cambium and phloem layers in the 1-year stem H5 of *Heptacodium myconioides*. Designations: ca – cambium; cc – companion cells; cr – crystals; cs – crystal sand; en – endoderm; pa – axial parenchyma; pf – phloem fibers; pr – pith rays; st – starch grains; sv – sieve tubes; xy – xylem. Scale bars 100 µm

A compact layer of sieve tube elements with companioncells 29.9 µm in diameter, positioned on the circumference (Figs. 16 and 20). The width of phloem was observed to be ca. 50% of that of wood layer. Sieve tube element with lateral sieve areas was also observed. Sieve tube plate were straight, positioned perpendicularly (Fig. 21).

Distinct layers of fiber sieve cells and phloem ground cells were present. Phloem fibers were very long, reaching up to 4000 µm. Parenchyma and ground cells of phloem rays were also visible. Those were overlaid by a tight layer of thick-walled cells of phloem fibers and sieve cells. The phloem fiber layer was compact, with large angular cells, positioned as 1–2 series of cells on the circumference of the layer of sieve cells. External cells were large, thick walled, sclerenchymatic. On the phloem side of the circumference there were crystals in the cells (Fig. 21).

**Fig. 22:**
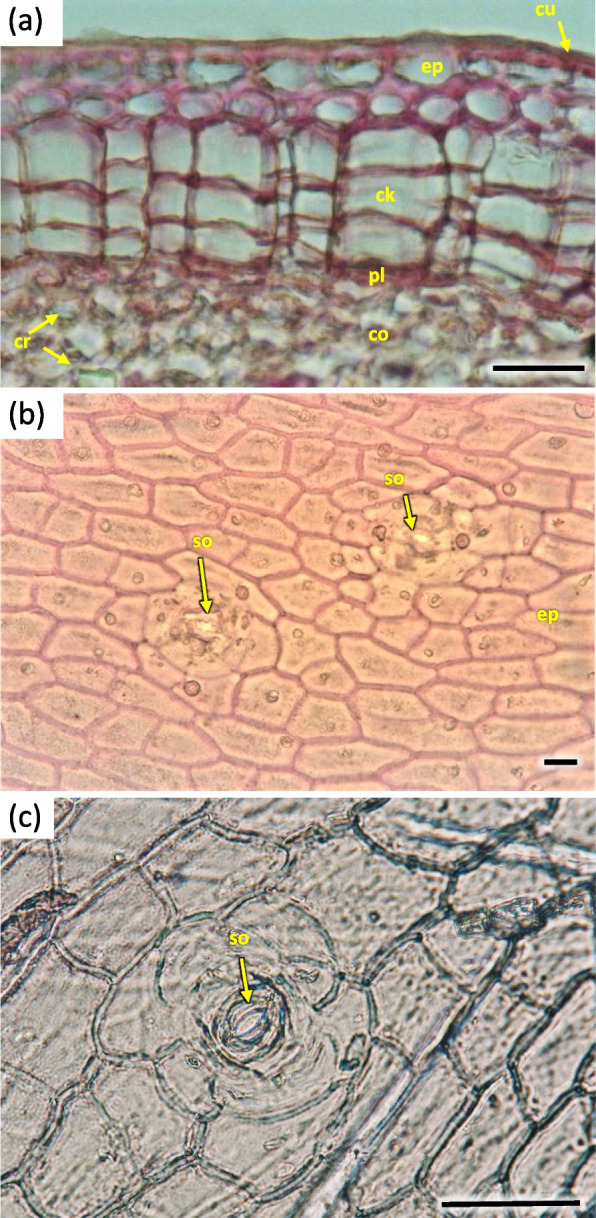
Transverse sections of periderm layer (**a**) and stomata of epidermis (**b**, **c**) in the 1-year stem H1 of *Heptacodium myconioides*. Designations: ck – cork; co – cortex; cr – crystals; cu – cuticle; ep – epidermis; pl – phellem; so – stomata. Scale bars 100 µm

**Fig. 23:**
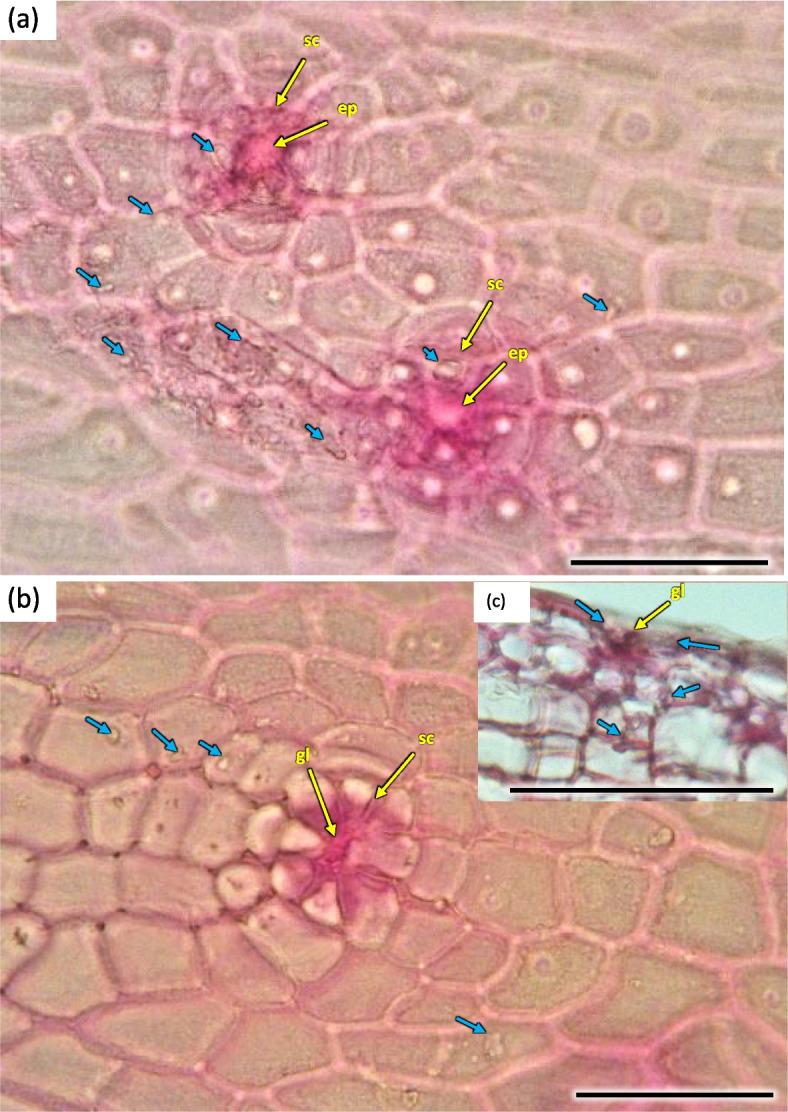
The part of epidermis in the 1-year stem H5 of *Heptacodium myconioides* with visible estuaries of secretory chambers (**a**, **b**) after maceration, and gland (**c**) in transverse section. Designations: gl – gland; ep – epithel; sc – secretory chambers; blue arrows – secretions. Scale bars 100 µm

The cortex layer on the circumference consisted of rather loosely positioned large cells, rich in starch granules (Figs. 16 and 19). The crystals and mineral inclusions were presented near the passage cells of endodermis (Fig. 16). Phellogen in stage H1 is composed of regular rows of cells and it remains active, diversifying to the inside into cortex cells and to the outside as cork cells. The outer layer of epidermis is composed of a single layer of cells on the circumference of stem (Figs. 16 and 19). Cork cambium is distinctly separated and active, it constitutes a base for 3–5 rows of cells arranged in layers (Fig. 22). A single cell thick layer of epidermis cells was observed, with visible nuclei in irregular cells and sparse stomas (Fig. 22) on the surface. The estuaries of secretory chambers and glands are visible (Fig. 23).

The anatomy of stems collected at H2 term was as above. Their cambium was narrow, regular, composed of bunches of 3–5 cells. On a small part of the circumference of the late wood cell walls of the last cells were not completely lignified, as evidenced by their blueish hue in safranin and fast green staining.

For Sa H3 and H4 no significant changes were observed. For H5 cell walls of wood cells were fully stained by safranin, which proves they were fully lignified (Fig. 16b). Cambium was present, although no signs of division or differentiation were seen along the circumference. Phelloderm was composed of distinct layers, with the cork layer strongly developed where a distinct detail was observed (Fig. 20).

### 2–6 years old stem anatomy (Sb)

**Fig. 24:**
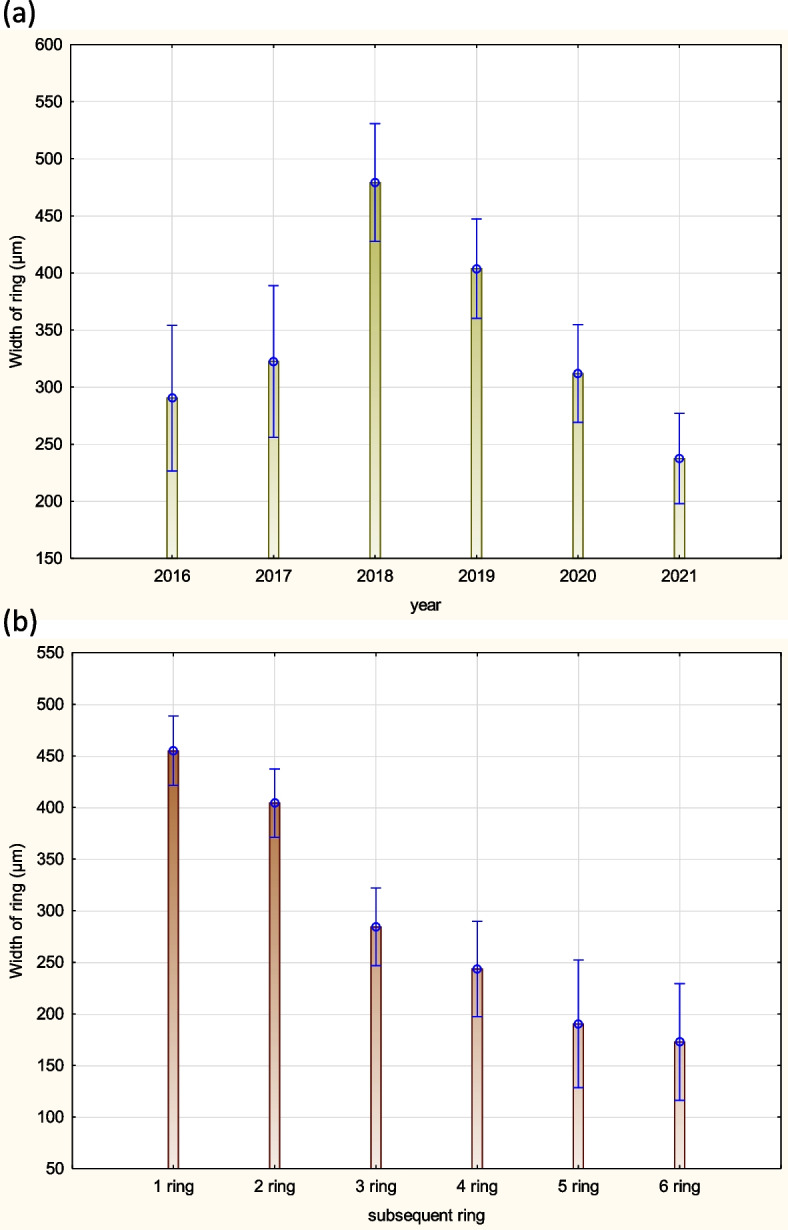
Width of rings characterized 2–6 years shoots of *Heptacodium myconioides* in fallowing years 2016–2021 (**a**) and in subsequent ring on cross sections (**b**). Vertical bars denote 95% confidence intervals for the mean of counts (one-way ANOVA)

**Fig. 25:**
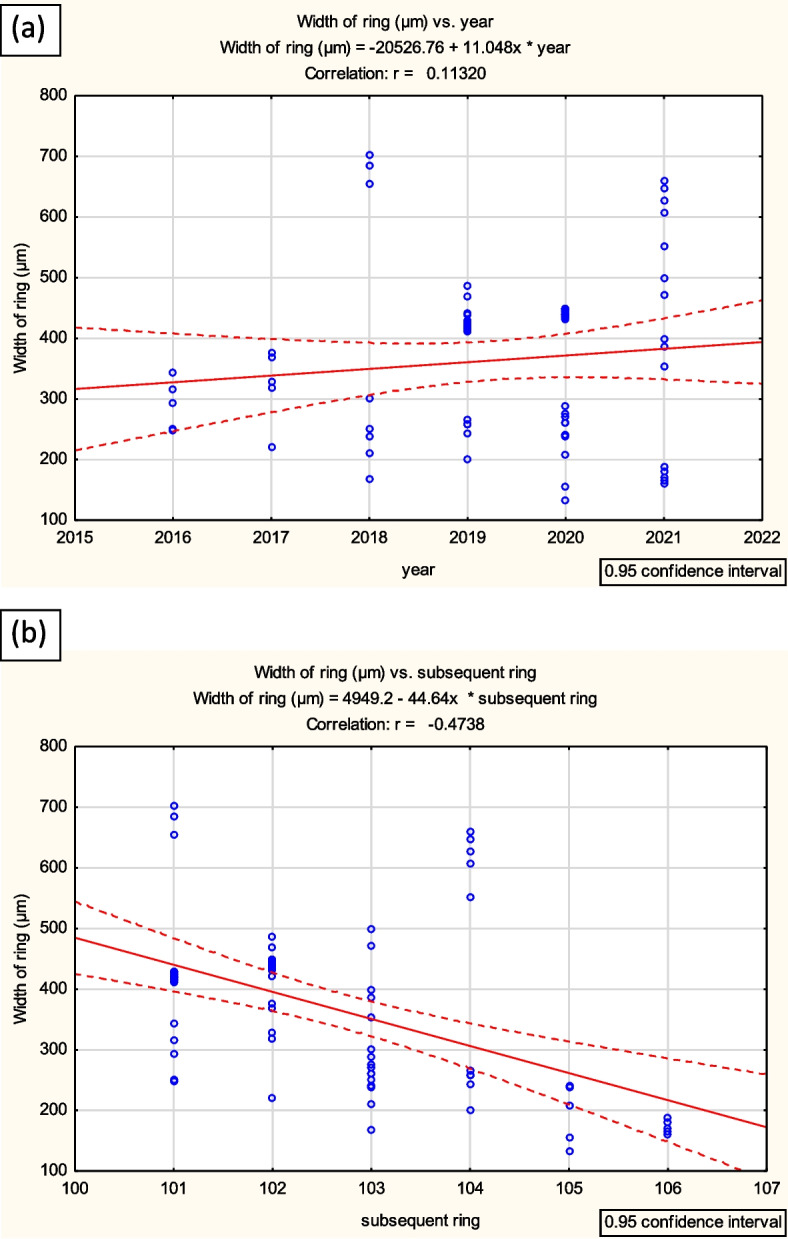
Regression analysis of the correlation residuals (**a**) between the width of the ring of annual growth (µm) of *Heptacodium myconioides* and the year of research; and (**b**) between the width of the ring of annual growth (µm) and the order of rings on the cross-section (subsequent ring 1–6) (analysis for the years 2016–2021)

The average width of annual rings of the 2–6-year-old stems is shown in Fig. 24. The width of consecutive rings varied, and in successive years of growth of the 6-year-old stem the width of annual ring decreased (Figs. 24 and 25).

**Fig. 26:**
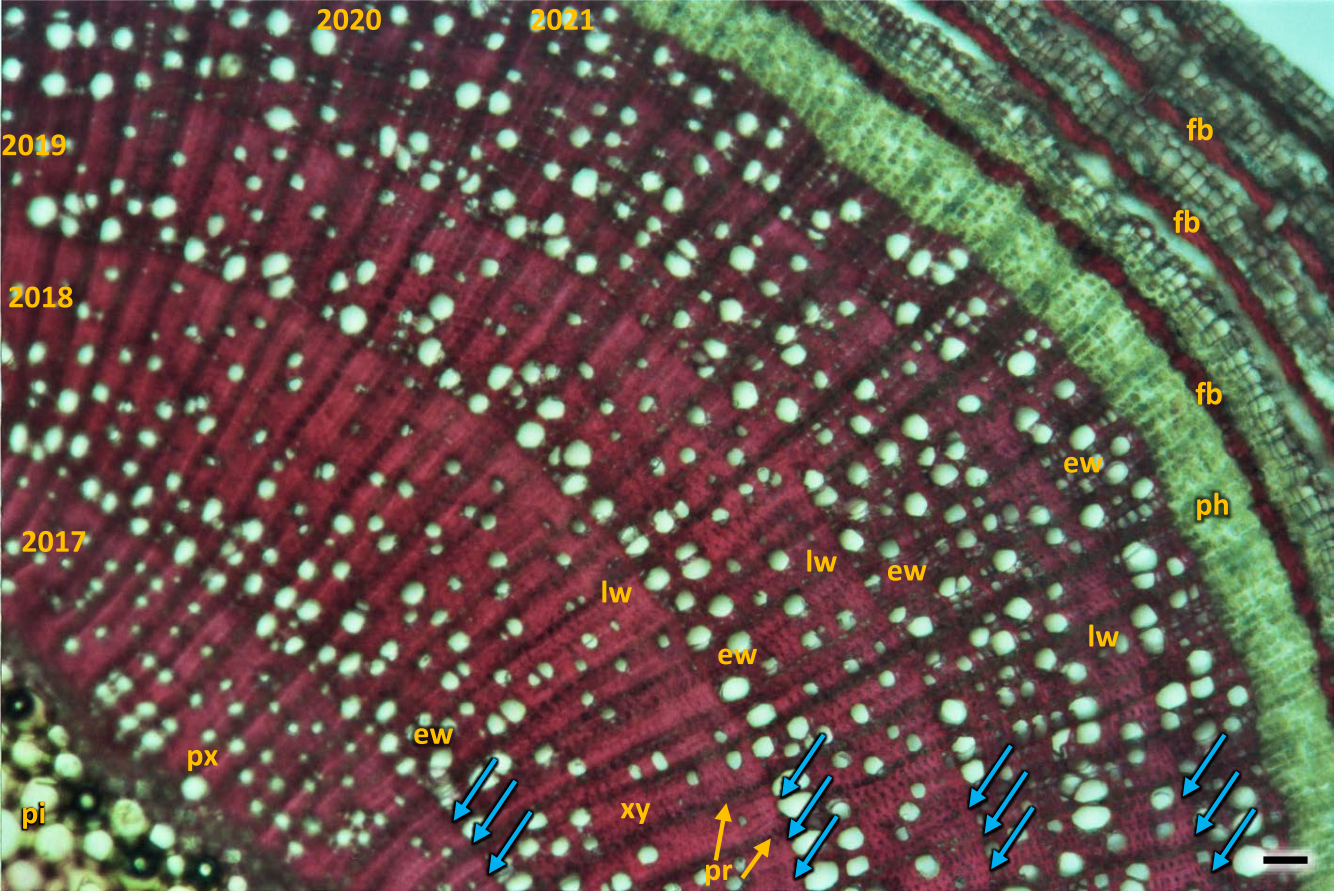
Transverse section of the *Heptacodium myconioides* the rings, wood semi ring porous. The border of annual rings was marked with blue arrows and described with year. Designations: ew -early ewood; fb – phloem fiber in band; lw – late wood; ph – phloem; pi – pith; pr – pith rays; px – primary xylem; xy – xylem. Scale bar 200 µm

Growth ring boundaries were distinct, the wood was semi-ring-porous with a tendency in 5–6 rings to diffuse-porous wood (Fig. 26). The early wood vessel diameter was wider than the diameter of late wood vessels (Table 2).

For Sb of 2–6-year-old H1-H5 in the radial cross-section the layer of early wood was less and less distinct in the consecutive years, consequently the difference between early and late wood is the least visible in the last ring of wood (Fig. 26).

Early wood was the widest and the most distinctly visible in the first ring of annual growth (Table 2). In the first ring it is regular, composed of parallel rows of 5–6 vessels. Bundles and rows of vessels were separated by bands of pith rays, usually consistent of a single row of cells, and reaching as far as cambium and phloem. On the inner side there was a wide layer of ground cells that were rich in starch. Solitary vessels on the outline were angular. The number of vessels per 1 mm^2^ in early wood differed in consecutive annual rings, as did the diameter and the lumen of the vessels (Table 2; Fig. 26). In the longitudinal section in 1 ring the walls of early wood vessels were spiral or annular. Helical thickenings were present throughout the body of vessel element or only in vessel element tails.

In the second annual ring the early wood was composed of loosely placed vessels, arranged in 2–3 regular, or more often irregular, layers. Vessels are arranged in a dendroid pattern. In the following third and fourth rings, the early wood consisted of only 1–2 rows of vessels. In the fifth annual ring in the late wood of 2018 the third ring of vessels was composed of xylem cells of large diameters, similar to those of early wood, albeit those were formed by the end of growing season (Fig. 26).

**Fig. 27:**
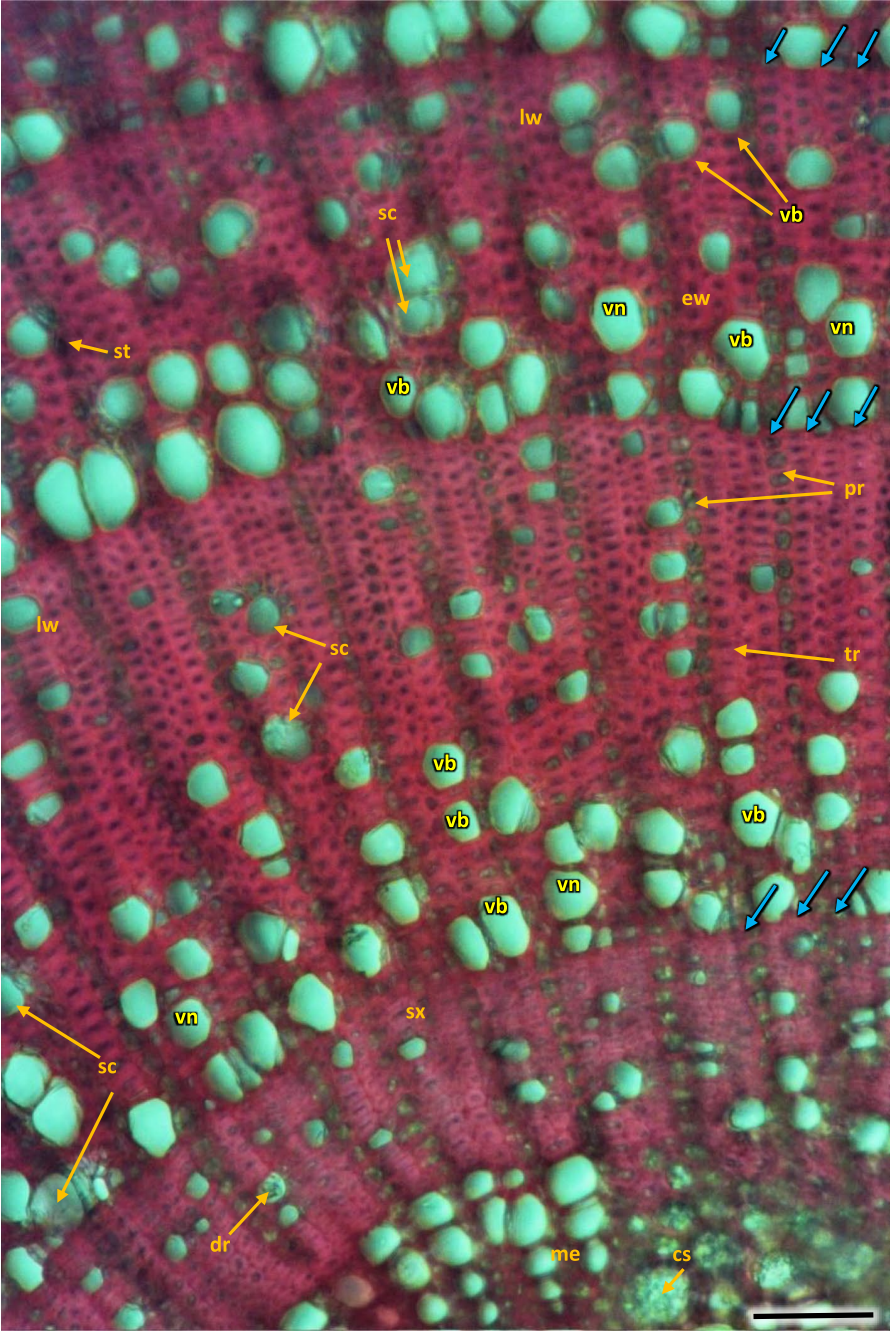
Transverse section of the 4 years old stem of *Heptacodium myconioides* – the rings, wood semi ring porous. The border of annual ring is designed with blue arrows. Designations: cs – crystal sand; dr – druses; ew -early wood; lw – late wood; me – metaksylem; pr – pith rays; sc – secretion cells; sx – secondary xylem; tr – tracheids; vb – vessels with contact pits; vn – vessels without contact pits. Scale bar 100 µm

The vessels in secondary xylem were rather sparsely distributed along the cross-section of the stem, albeit in 2–6 years the number of vessels per 1mm^2^ was high, although variable and different (Fig. 26, Table 2). Vessels were partly solitary, partly in radial multiples of 2–4, or very small clusters. Solitary vessels outlines were angular (Fig. 27). The width of late-wood layer and the diameter of vessels changed in the following annual rings (Table 2).

**Fig. 28:**
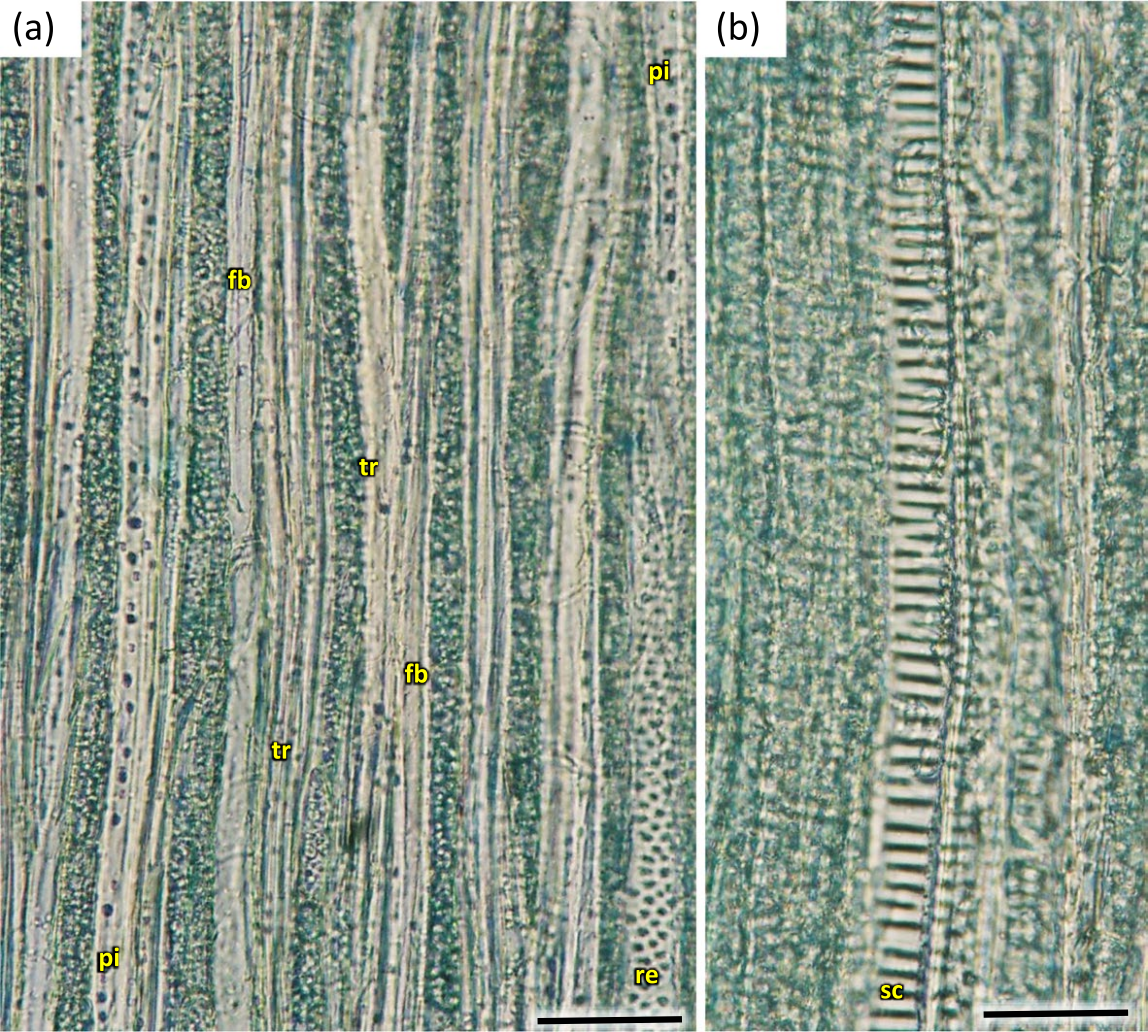
Longitudinal radial section of the 5 years old stem of *Heptacodium myconioides* – the vessels (**a**, **b**). Designations: fb – fibres; re – reticulate thickening of vessel; pi – pitted thickening of vessel; sc – scalariform vessel; tr – tracheids. Scale bars 100 µm

**Fig. 29:**
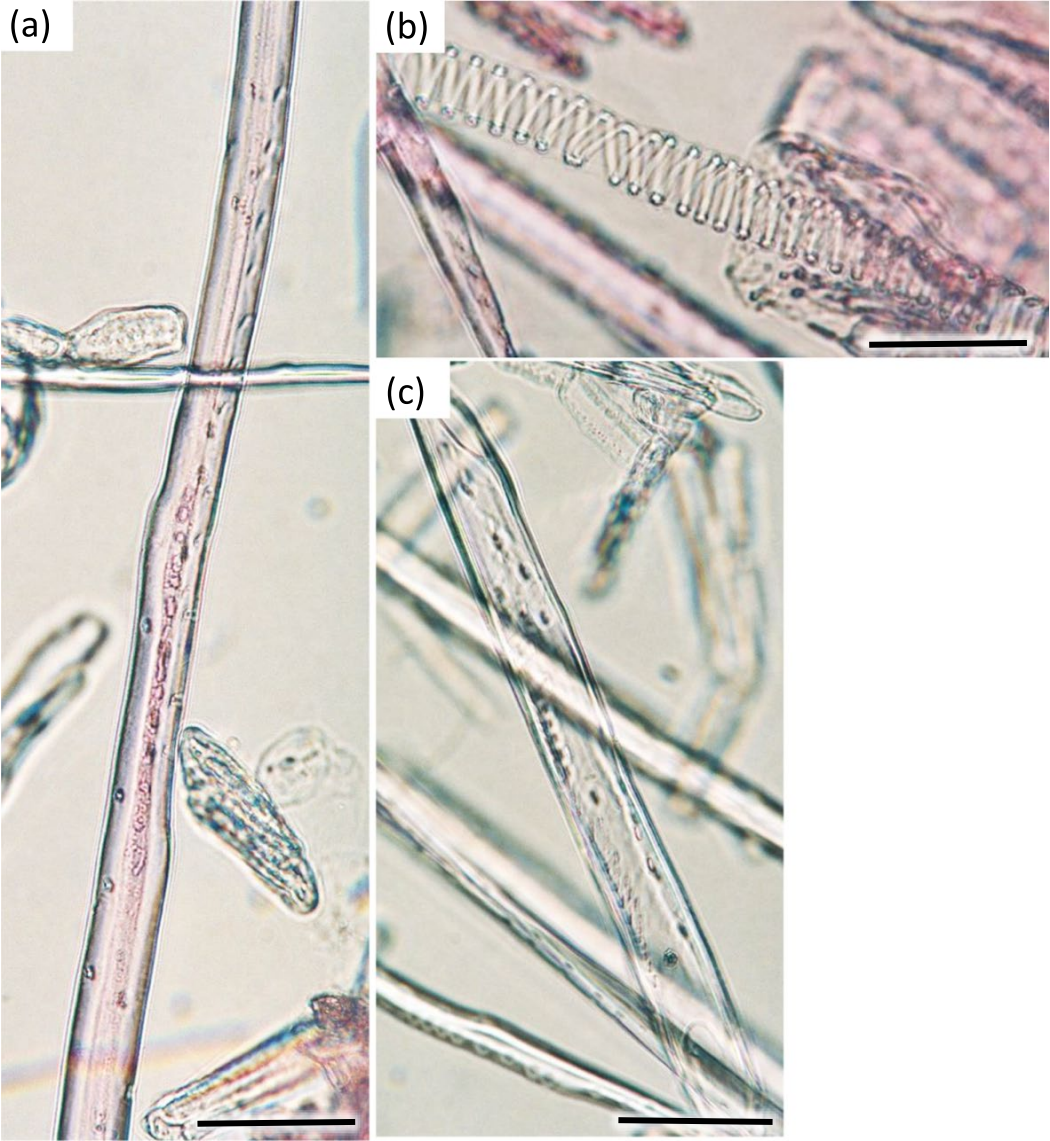
The a 4-year stem in *Heptacodium myconioides*, after maceration: **a** wood fiber with inclusion; **b** spiral thickening of vessel; **c** the vessel pitted thickening, simple perforation plate. Scale bars 100 µm

**Fig. 30:**
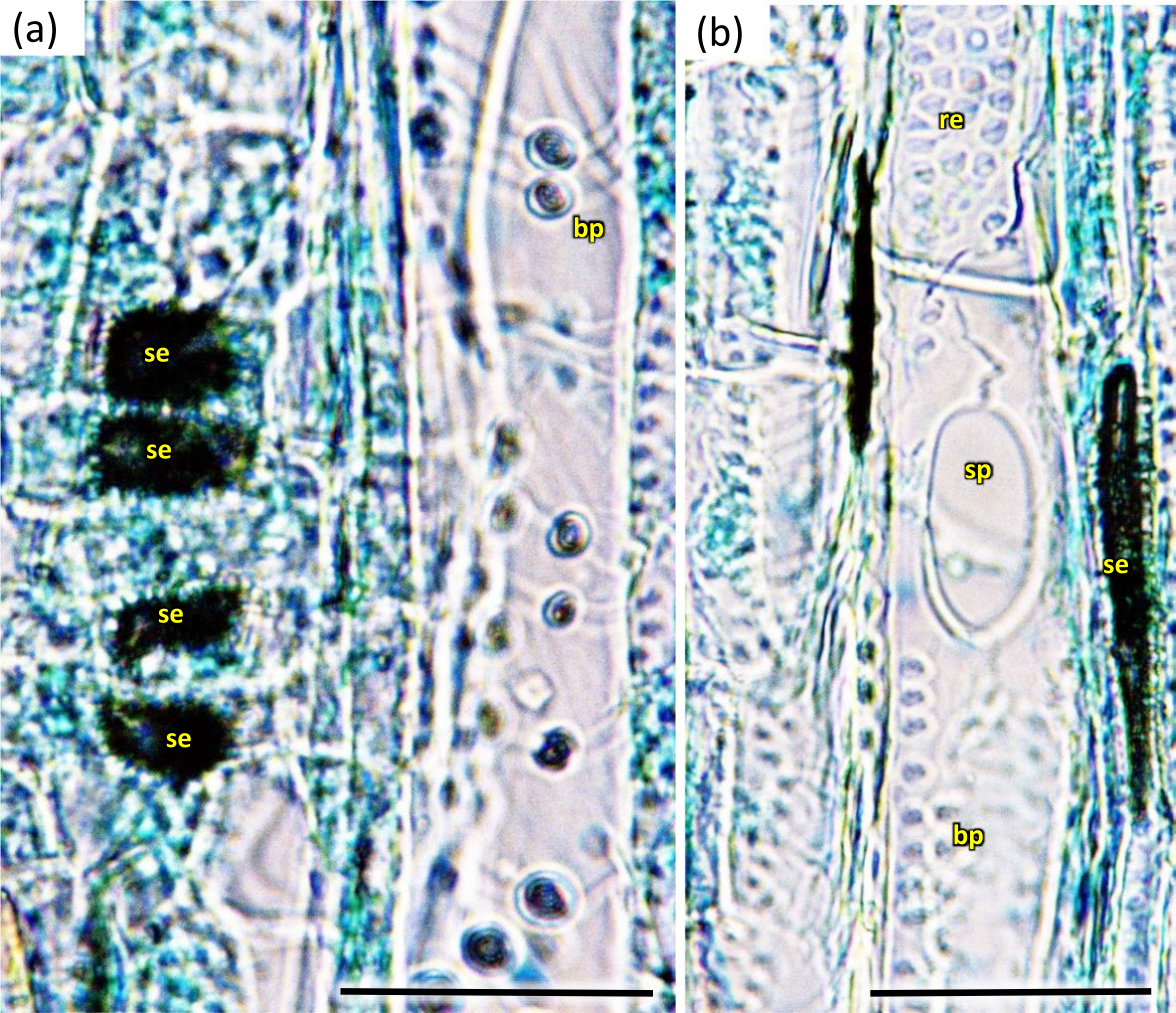
Xylem vessel structure on longitudinal tangential section of the 5 years old stem in *Heptacodium myconioides* (**a**, **b**). Designations: bp – bordered pitted walls of vessel; re – reticulate thickening of vessel; se – secretions in pith ray parenchyma; sp – the large pits resemble perforations. Scale bars 50 µm

**Fig. 31:**
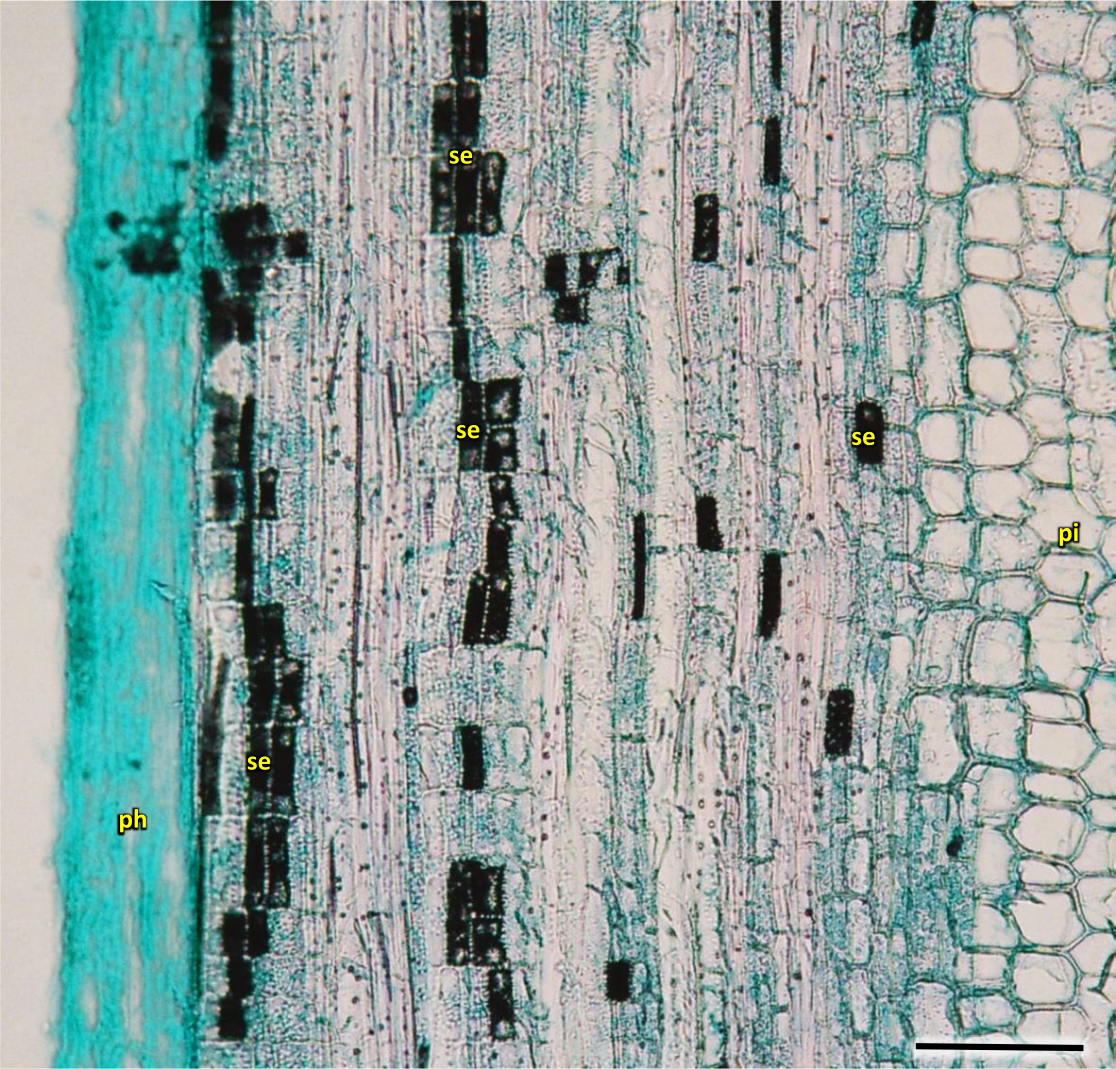
Longitudinal radial section of the 3 years old stem in *Heptacodium myconioides* with visible secretions in ray parenchyma. Designations: pi – pith; ph – phloem; se – secretions in pith cells. Scale bar 500 µm

**Fig. 32:**
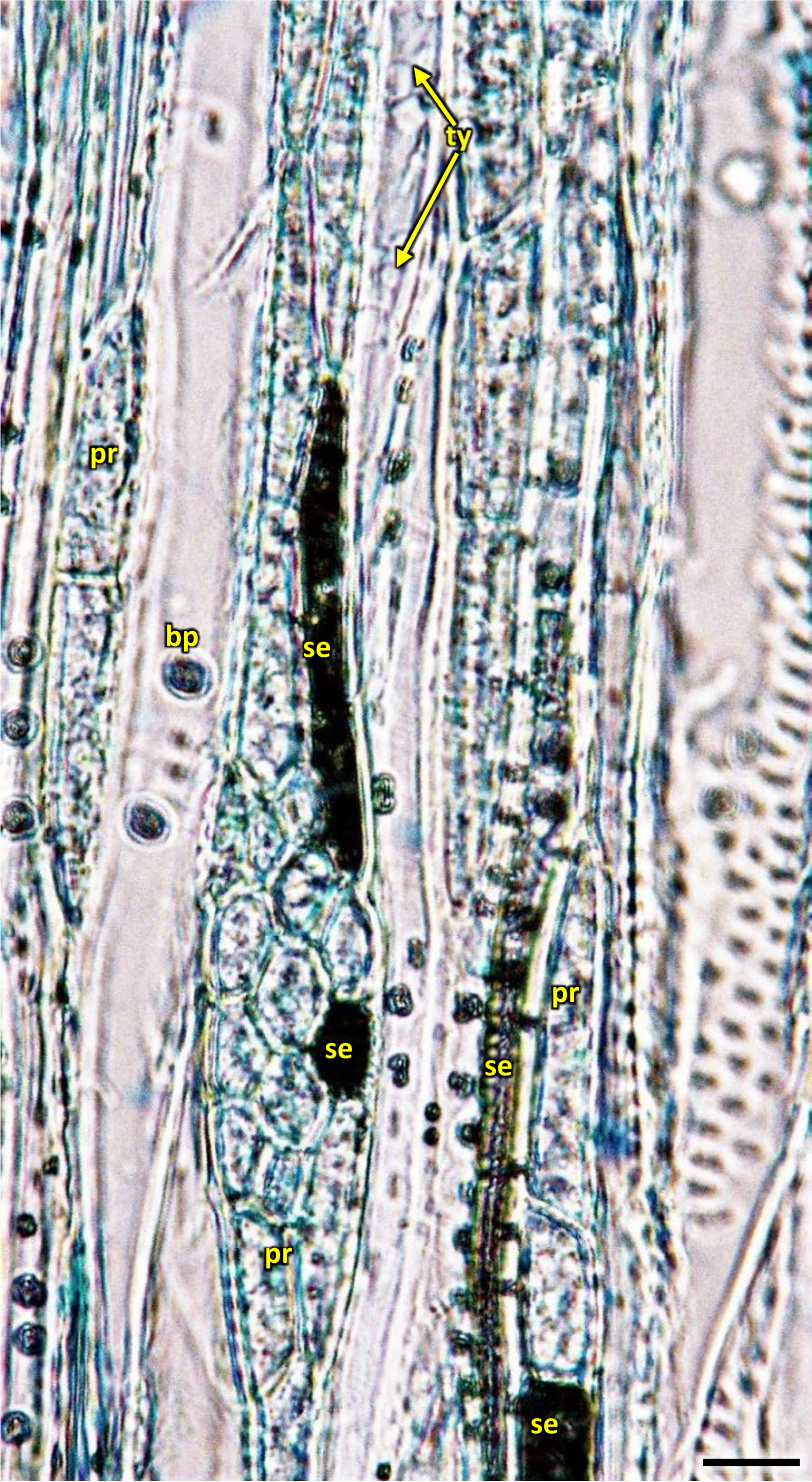
Xylem and pith ray in longitudinal tangential section of the 3 years old stem in *Heptacodium myconioides*. Designations: bp – bordered pit; pr – pith ray; se – secretions; ty – tyloses. Scale bar 50 µm

**Fig. 33:**
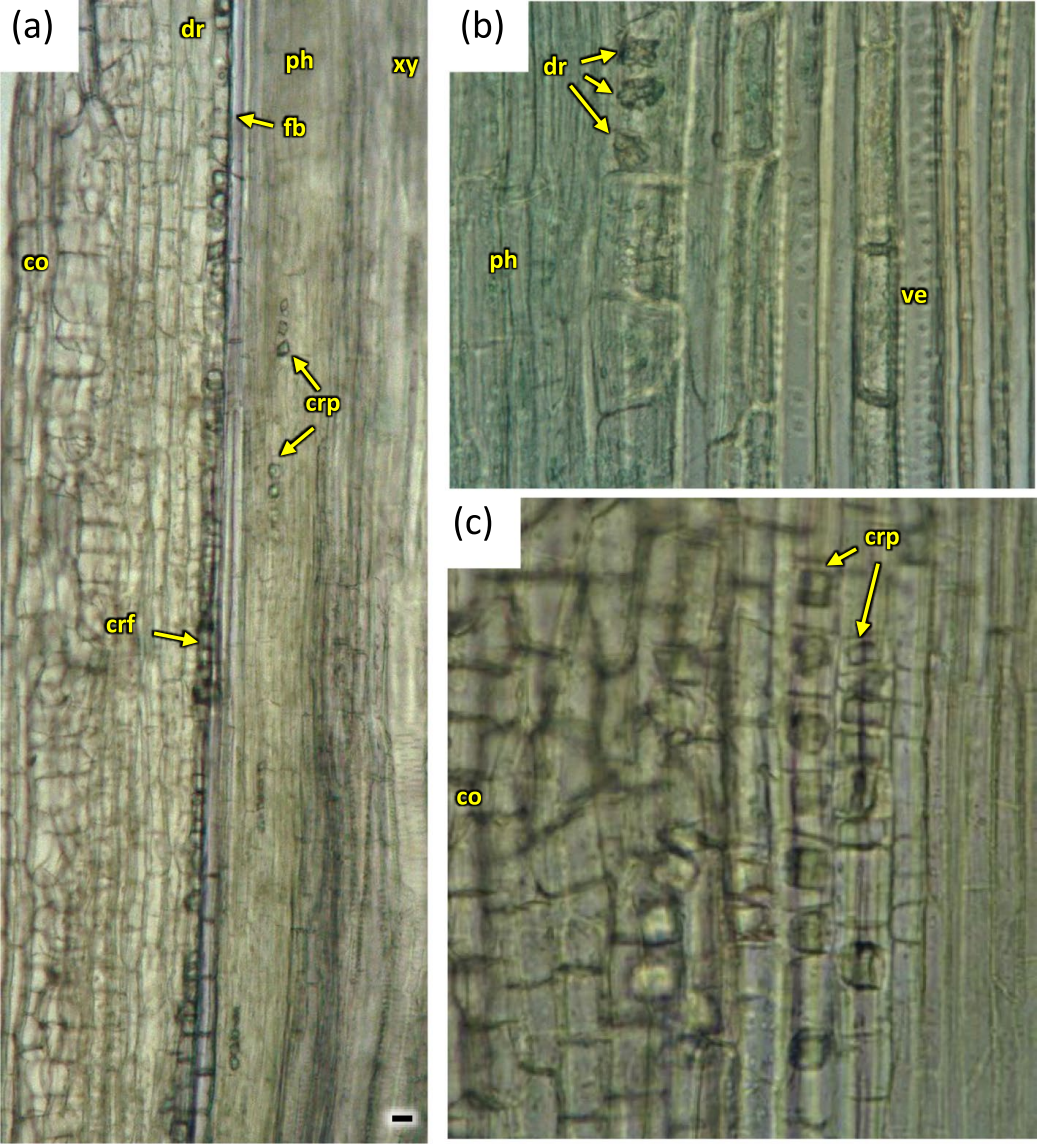
Crystals visible on longitudinal radial sections of the 3 years old stem in *Heptacodium myconioides* (**a**, **b**, **c**). Designations: co – cortex; cr – crystals; crf – crystals outside band of phloem fibers; crp – row of prismatic crystals in phloem tissue; dr – druses; fb – band of phloem fibers; fi – fibers; ph – phloem; tr – tracheids; ve – vessel; xy – xylem. Scale bars 100 µm

In vessels of late wood were also scalariform walls, less frequently reticulated and pitted. The vessels were joined straight or less frequently scalariform (Fig. 28). Simple perforation plates or scalariform with 2–3 bars. Fibers thin- to thick-walled, helical thickenings in ground tissue fibers. Those were on average 600–1200 µm long, albeit their length may reach up to about 4000 µm. Intervessel piths were scalariform to opposite. Axial was absent in parenchyma or extremely rare. Rays with procumbent, square and upright cells were mixed throughout the ray (Figs. 28, 29 and 30). The crystals and secretion cells were visible among the fibers (Figs. 30, 31, 32 and 33).

**Fig. 34:**
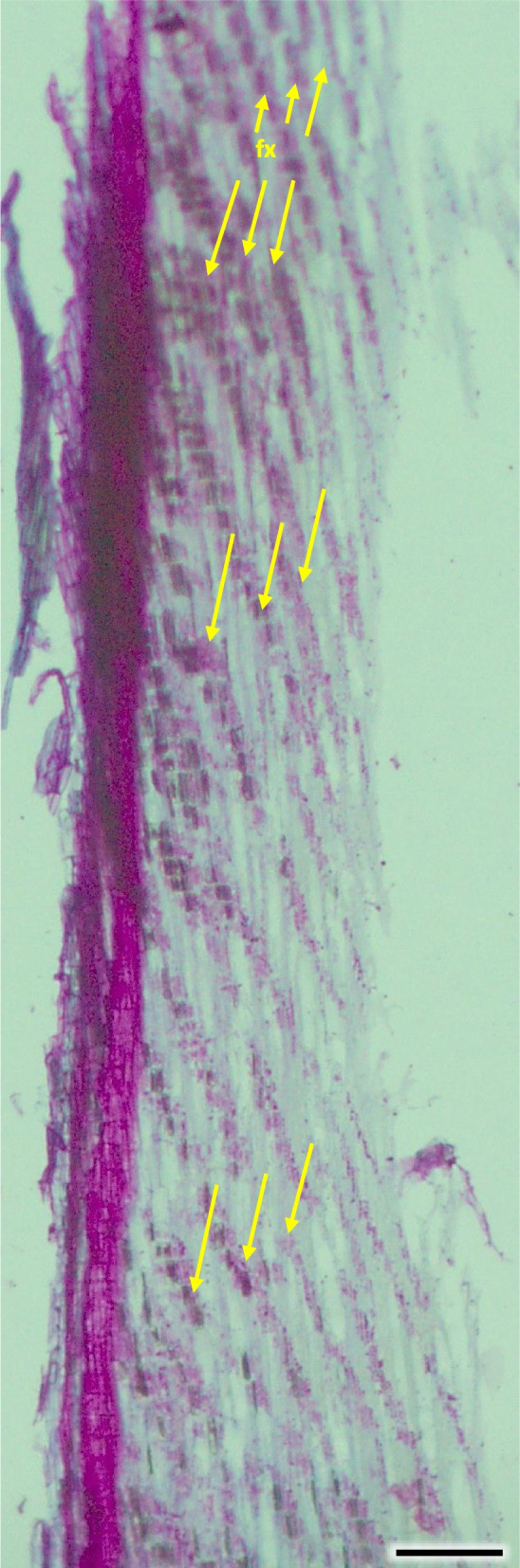
Corrugated fiber (yellow arrows) in *Heptacodium myconioides*, longitudinal radial section. Designations: fx – fibers. Scale bar 500 µm

Pith cells were irregular and big (Figs. 26 and 27). The diameter of pith was similar for stems of different ages. In the first ring 2–3 rows of vessels were divided by 1 row wide medullary rays. From the place of formation of first bundles and vessels there were 2 to 3 rows wide pith rays, running continually from pith ground cells to phloem (Fig. 26). Consecutive medullary rays 1–2 seriate form in the new annual rings, most often in the latewood layer, growing together with the increase in stem thickness and continued to extend reaching to phloem. There were many starch nodules visible in the rays (Figs. 26 and 27). The cross-section of the radial surface showed regular strips of wood fibers, which can be initially defined as the phenomenon of corrugated fiber. On the examined sections, the waviness slope on the surface of the radial section (α) was visually 35–45°, the fibrous wavelength (λ) is 120–150 µm (Fig. 34).

**Fig. 35:**
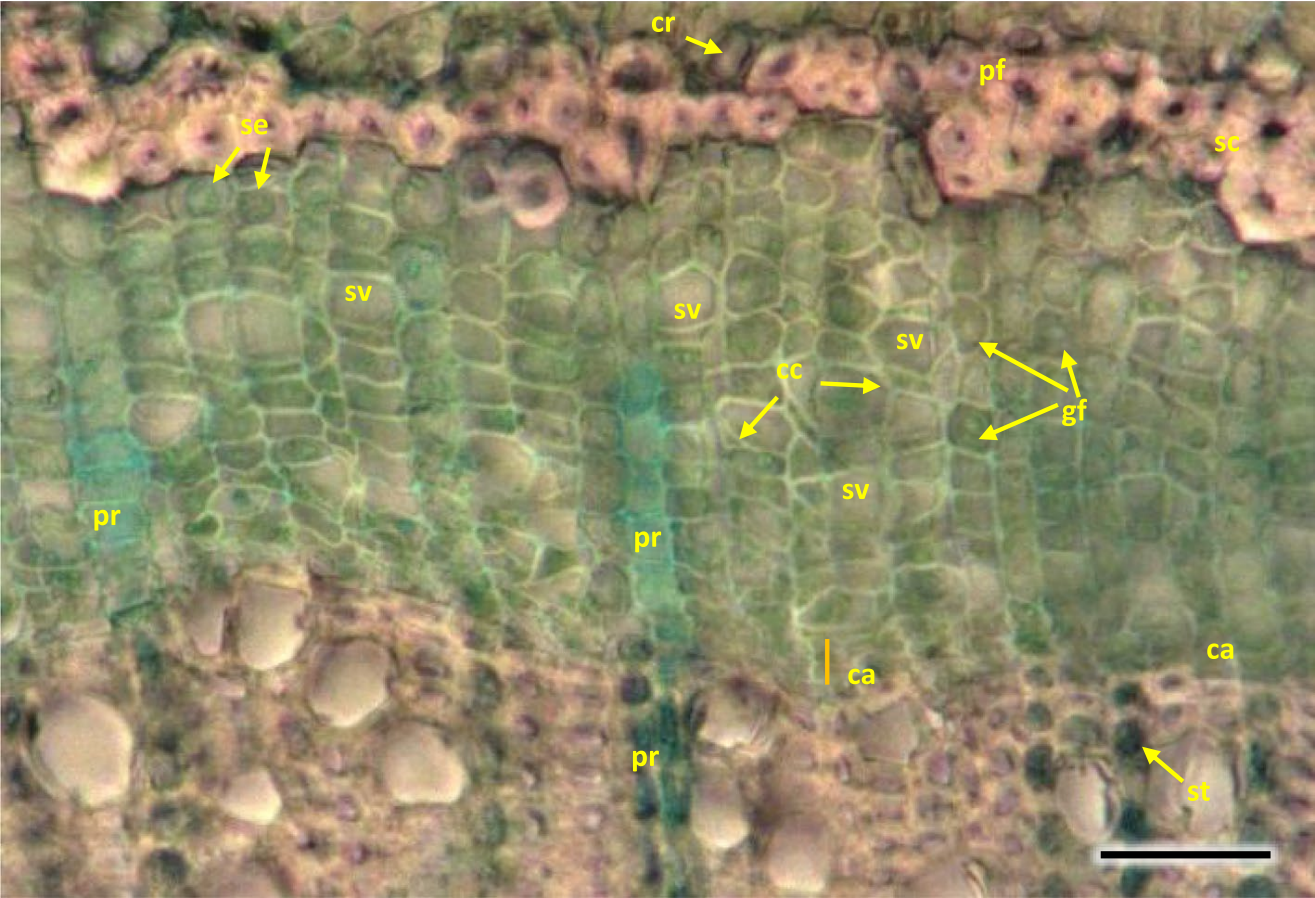
Transverse section of the phloem last year (2021) in 5 years old stem of *Heptacodium myconioides*, Designations: ca – cambium; cc – companion cell; cr – crystals; gf – gelatinous fibers; pr – pith ray;sc – sclereid fiber cells of phloem band; se – secretions; st – starch grains; sv – sieve cells. Scale bars 50 µm

**Fig. 36:**
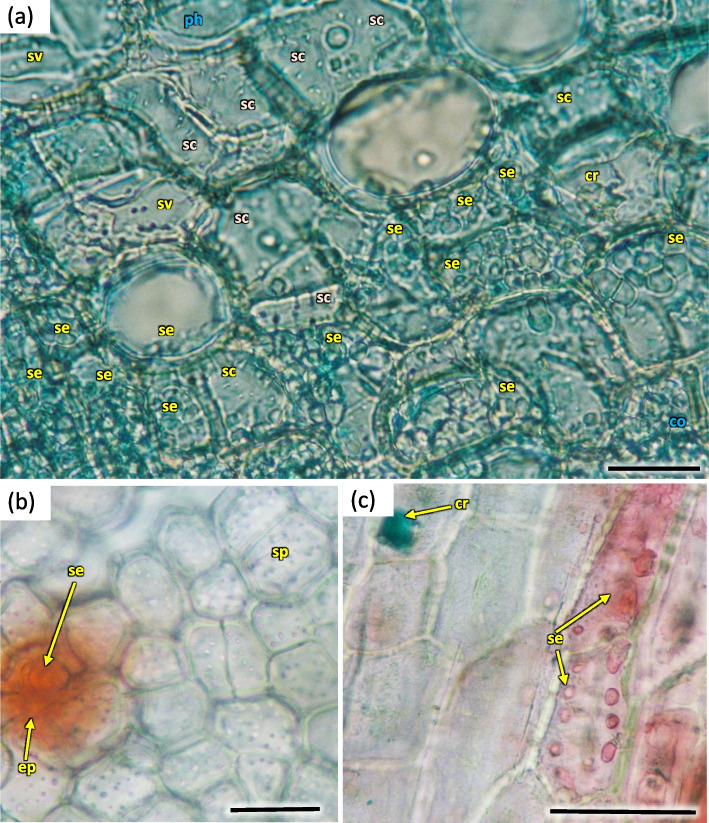
Transverse section of phloem last year (2021) in 5 years old stem of *Heptacodium myconioides*, (**a**) and secretion cells (**b**, **c**). Designations: cr – crystals; co – part outside fibers band; ep – epithel; ph – phoem; sc – sclereid fiber cells of phloem band; se – secretions; sp – sieve plate; sv – sieve cells. Scale bars 50 µm

**Fig. 37:**
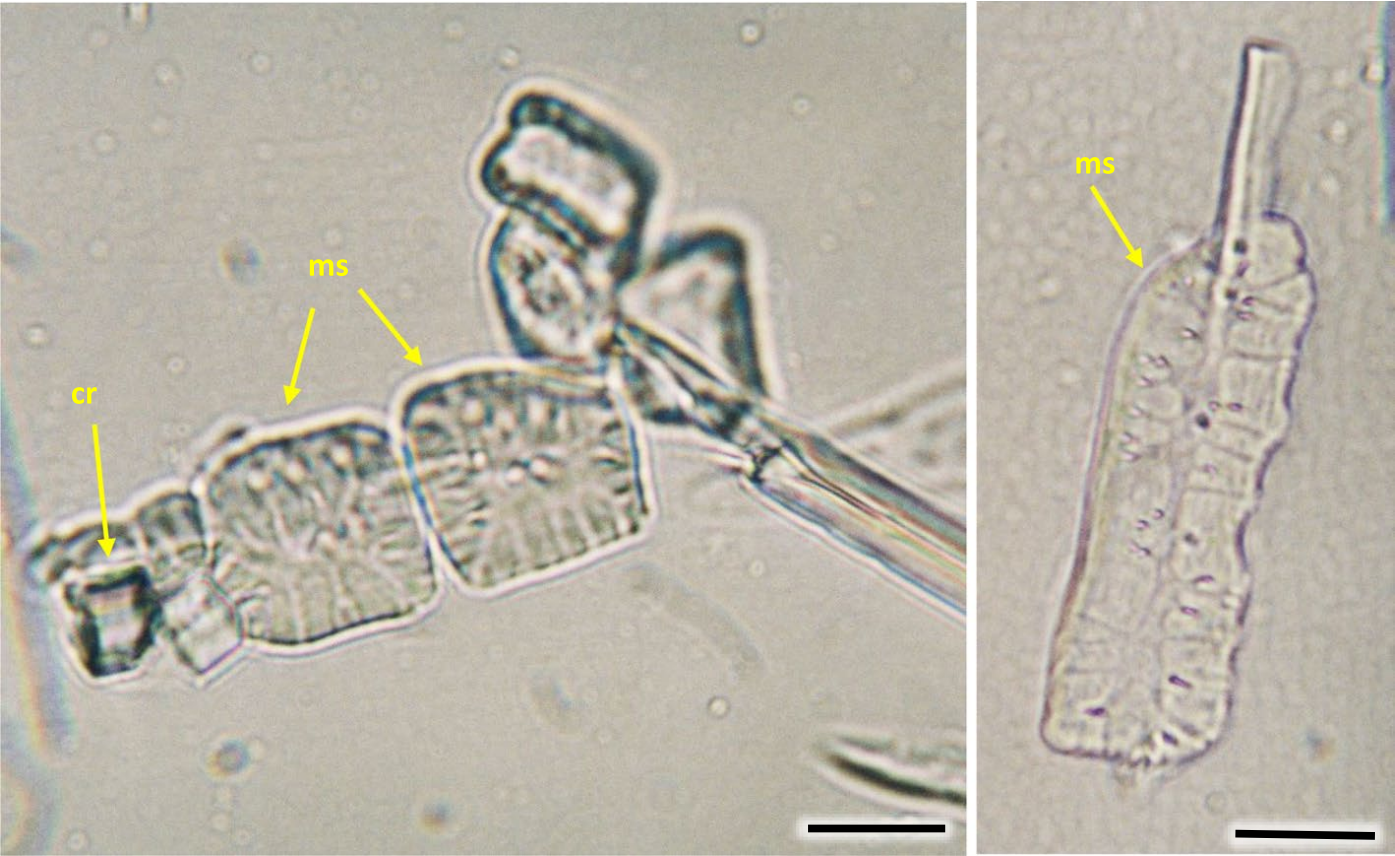
Sclereids in *Heptacodium myconioides*, after maceration. Designations: cr – crystals; ms – mature sclereids. Scale bars 50 µm

The phloem layer was thin. There was a distinct layer of sieve tubes with companion cells, sieve fibers, phloem ground cells and ground cells of pith rays. It was closed by a dense layer of thick-walled phloem fibers. The anatomy of phloem was like in the annual stems, albeit with longer phloem fibers (Figs. 26, 27 and 35). Secretory cells were present, which contained crystals of calcium oxalate and other secretions (Figs. 33, 35 and 36). Sclerenchymatic cells were also present. On the other hand, the sclerenchyma layer along the axis of the shoot has arranged schizogenic spaces of the secretory chambers forming the epithelium (Figs. 26, 27, 33 and 37).

Cambium consists of a layer of 3–4 to 5–6 cells and shows activity and cell division up to H1 stage. Tracheids were not yet fully lignified in the last ring of late wood cells in the stems of H3 stage.

**Fig. 38:**
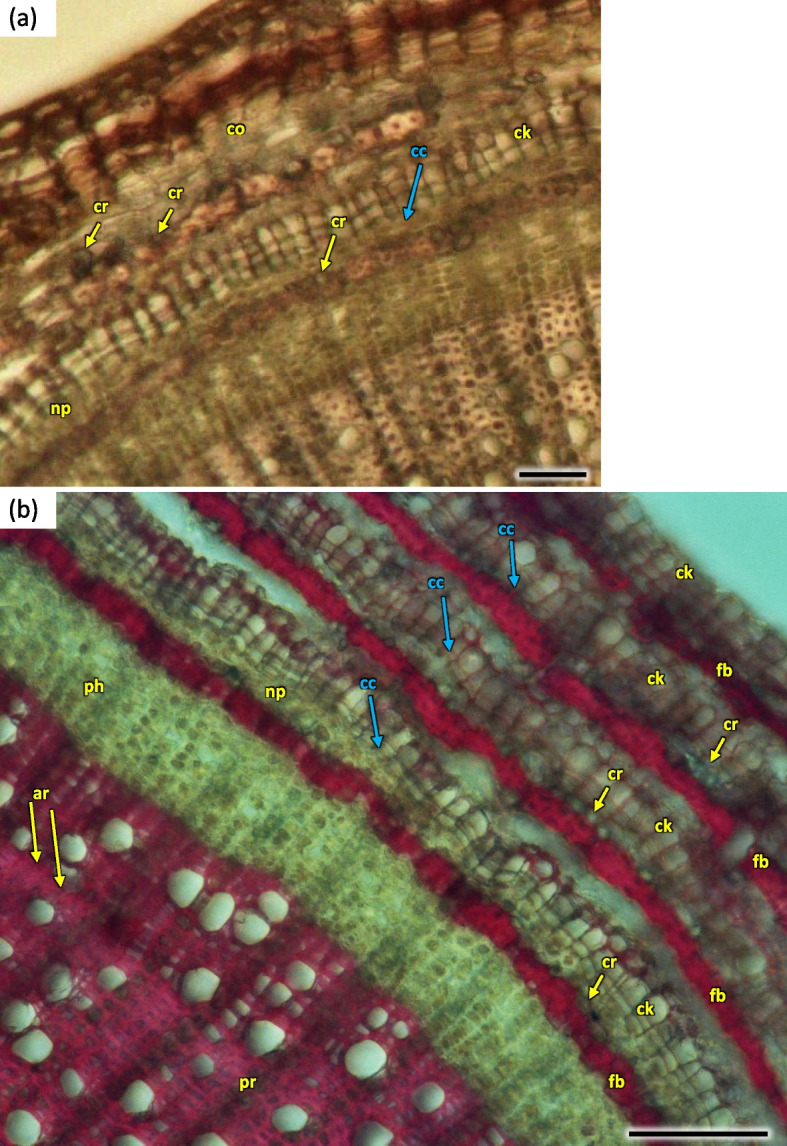
Transverse section of phloem and bark in two (**a**) and 5 years (**b** the last layer of bark absent) old stem of *Heptacodium myconioides* H2. Designations: ar – annual ring; cc – cork cambium; ck – cork; co – cortex; cr – crystals; fb – sclereid fiber cells of phloem band; np – nonconducting phloem; ph – phloem; pr – pith rays. Scale bars 200 µm

Layers of phelloderm were clearly visible. Cork-forming cambium is clearly distinguished, forming a layer of 3–5 superimposed cells, which differentiated by H4 stage (Figs. 14 and 15). Rather large, regularily organized, cortex cells of 1- and 2-years old shoots were deposited to the inside of the stem. Parenchymal cells were sparse, some contained small prismatic crystals. Smaller and slightly more compactly spaced cork cells were differentiated to the outside. The last outside layer was formed by regular epidermis cells, in older stems flaking of the bark was observed (Fig. 38a). The outer bark of older shoots is formed by sequent periderms, which enclose part of the nonconducting phloem, constituting a rhytidome. The outer layers consist of the secondary phloem, band of phloem fibers and the outer bark (Fig. 38b).

The last ring of tissues of Sb in H2-H5 stages had similar structure and were lignified to a similar degree. Cambium still shown similar, local activity in H2 and H3 stages. Woody tissues were completely lignified by 3.11. (H4).

## Discussion

Observations carried out in this work allow us to identify *Heptacodium miconioides* as a species tolerant to climatic conditions of Central Europe, zone 6B [44]. Despite the observed delay in vegetative growth initiation after cold winter months in Wrocław in 2013–2014, the flowering date was similar at both research sites. In Warsaw however, where the winters during the years preceding our study were mild, *Heptacodium miconioides* begun leafing (BBCH 01) significantly earlier and simultaneously the leaf cover on bushes stayed for up to 44 days longer in total than in Wrocław (Figs. 13 and 14). Climate warming contributes not only to reducing of frost events and lengthening of growing season in the Northern Hemisphere [41]. In the years 1982–2020 last frost events occurred in Poland 7–14 days earlier than in the preceding decades [40]. The lengthening of growing season most likely favors the deposition of wider layer of annual rings in *Heptacodium miconioides*, despite the fact that for 2–6-year-old stems a tendency of decreasing width of annual rings was observed in the subsequent years of growth of the stem (Figs. 24 and 26). It is possible the observed increase is caused by the late activity of cambium. Annual ring increase in the year 2018 was wider than expected most likely because of observed increased precipitation during the winter months and consistently low average temperature of February and March (Figs. 5 and 6), as it benefits the soil water resources by inducing slow melting of snow cover. It would confirm the results of [24], that xylem cell development is influenced by precipitation.

However, despite a tendency of reducing the total number of frost days per year, the number of days with minimum temperature < 0 °C nonetheless increased in many areas in Europe [41]. In the case of *Heptacodium miconioides* the lengthening of leafing overlaps with early phenological stages, such as leaf unfolding (Figs. 10 and 11). Similar results were obtained for nine woody species where the start dates of spring and summer phenological phases occurred earlier with time, while the beginning of autumn and winter phenological stages were delayed [62]. Despite the differences in the occurrence of inflorescence of flowers visible, and beginning of heading (BBCH 51), which seems to be connected to the formation of 4–5 leaf pairs on the generative stems Sa, the beginning of flowering (BBCH 61) happened at a similar time at both sites. It could suggest that other factors besides the weather influence flowering in plants [62] and physiological research in this field is needed. Moreover, the phenological stage when shoot development is completed, but the leaves are still green (BBCH 91) occurs at a similar time as stage of inflorescence of flowers visible, beginning of heading (BBCH 51) (Figs. 13 and 14). We presume that the formation of flower buds in *Heptacodium miconioides* occurs in the growing season preceding flowering, as it does in numerous other trees and shrubs of temperate zone. Among them is for example the apple tree, where the flowering spur shoot is a short shoot in which extension growth is limited to the production of a rosette with few leaves. The spring bloom in apple tree is the end of reproductive development that begins with floral initiation in the preceding summer and is broken by dormancy period in the winter [63]. Among other trees and shrubs which flower in a similar way among the *Caprifoliaceae* family, which is represented also by *Heptacodium miconioides*, *Diervilla lonicera* and *Weigela floribunda*, for example.

As was shown in the previous research, timing of the dynamics in vessel formation is different between ring porous peduncular oak and European ash and depends on leaf phenology [30].

Annual stems Sa possess primary growth typical for woody dicots [63]. At the sample collection times H1-H5 vascular bundles form a closed ring structure characteristic for mature stem in trees [64].

Analyzing the stem cross-section, some differences in the rings structure could be observed in this paper, compared to Jacobs et al. [14]. Wood of *Heptacodium miconioides* was defined as diffuse-porous [14], because of similar diameter and even distribution of vessels in early and late wood [58]. Stems investigated by Jacobs et al. [14] were < 2 cm in diameter, where we investigated much thinner stems: the annual ones were circa 2–4 mm in diameter and the perennial ones up to 1 cm. In our research we have observed the appearance of significant traits of diffuse-porous wood no sooner than in the 4 to 5 years old perennial stems, and none in annual ones. Despite this, we think that wood semi-ring-porous is the most appropriate classification. This is also supported by small difference between the diameters of vessels in early and late wood, serial arrangement of vessels in early wood and a much greater number of other xylem elements in late wood (fibers, xylem, parenchyma cells and tracheids) (Figs. 26 and 27). The differences may have been caused by the differences in stem age, as well as in those caused by climatic conditions. Jacobs et al. [14] do not list the sites of plant material collection, it is therefore difficult to compare them to our sites.

Annual stems as well as 1–4 rings in a perennial stem up to a few years old show a notable difference in the number of vessels in early and late wood, as well as in the visibility of the borders of annual rings. It is in all likelihood associated with intense growth of the young stems along the length, as well as to flowering in the case of annual stems. Stems older than 6 years were not investigated. Stems possess the characteristics of softwood; the tissues are alive and carry out their functions in transport. The pit of perennial stems is also alive. For 2018 a band of vessels deposited by the end of xylem growth period was observed. It could indicate that cambium activity is high during the flowering period. It was showed in the research of Patel et al. [24], that the peak cambial cell division and rate of xylem differentiation in drum-sick tree (*Moringa oleifera*) is associated with rainfall during the monsoon period. Low temperature were correlated with this phenomenon. However, the cambial activity and vessel differentiation in *Heptacodium miconioides* had to be due to another reason because there was no high precipitation in the period of July–October of 2018 (Fig. 5).

The cross-section of the radial surface shows regular strands of wood fibers, which can be preliminarily defined as the phenomenon of corrugated fiber [64]. The filamentous nature results from uneven deposition of fiber by cambium, however those are quite complicated phenomena [64], hence it requires confirmation and further analysis on bigger sample sets and older stem samples. It was shown that on the examined sections, the waviness slope on the surface of the radial section (α) was visually 35–45°, the fibrous wavelength (λ) is 120–150 µm.

In our investigations of woody tissue, we found similarities, but also differences to results published by Jacobs et al. [14]. It was shown that in the first 1–6 years of the life of a stem the growth rings boundaries were distinct. Jacobs et al. [14] wrote that growth ring boundaries were distinct to indistinct, which could have resulted from the age of the stems, as those had slightly bigger diameter < 2 cm, than those 1–6-year-old stems that we researched, measuring up to1 cm. The growth ring boundaries in *Heptacodium* were designated by differences in vessel density and fiber thickness, as in other woody plants [65–67]. The wood was described as diffuse-porous [14]. However, the wood we observed showed traits of semi-ring-porous, due to much higher density of vessel distribution and their much higher lumen in early wood. The vessels perforation plates are simple, but a single scalariform perforation could be in primary xylem [14] and such patterns were also often observed in secondary wood. The vessels can be angular and mostly solitary in late wood. The first pith rays are 2–3-seriate, the next in the secondary wood are mostly 1-seriate.

Jacobs et al. [14] observed that some ray cells contain one large prismatic crystal per cell. To the contrary, the crystals we observed in cortex parenchyma probably were connected to the secretory chambers of the cells of the phloem sclerenchyma layer. The epithelia of sclerenchyma secretory chambers located along the stem implies some compounds are secreted to the chamber. As secretory chambers do not form in tissues forming single layers [64], it is more likely they are connected to the phloem. Solving of this problem requires further research, however.

The cambium activity in *Heptacodium miconioides* finishes concurrently with the ending of flowering, and leaf falling. The process of prewinter lignification of tissues ends by the late leaf fall phase. Patel et al. [25] established, that for evergreen species from warm climatic zones (Pakistan, India) xylem cells development is affected by rainfall and rarely by temperature. Additionally, it was found that for *Moringa oleifera* the walls of vessels are lignified first, and the ray cells are lignified last [25]. For deciduous species *Castanea sativa,* flowering in May, the formation of early wood was completed by the end of May and 2–3 weeks earlier than early phloem. However, wood and phloem production terminated in the middle of August. The wood ring was completed by middle October and phloem ring by beginning of October [23].

## Conclusions

*Heptacodium miconioides* is not a very well researched species. Therefore, our research could be considered preliminary. The value of such a study is potentially high, as the ecological status of *Heptacodium miconioides* is currently unknown, while it is highly noteworthy as an ornamental plant, especially viable for planting in big agglomerations, as it poses no threat of becoming an invasive species. Such a way of utilizing endangered species contributes to their preservation in situ. The ornamental plants are important for both gardens and urban green spaces, however their tolerance for multistress growth conditions is necessary. Our phenological observations carried out in Wrocław and Warsaw indicate that *Heptacodium* has a significant potential for wider application, additionally highlighting the benefit of its late blooming and flowering.

Late and long flowering period could result in a prolonged cambium activity, despite the fact that vegetative growth terminates as soon as the beginning of June, similarly to many other deciduous species of the temperate zone. However, the lignification of tissues before winter ends no sooner than by the late leaf fall stage.

The growth ring boundaries are distinct, the wood semi-ring porous, early vessels in tangential bands, late wood in a radial pattern. There were common small clusters of early wood vessels, late wood vessels were partly solitary, partly in radial multiples of 2–4, or in very small clusters. The growth ring boundaries were distinct, the wood semi-ring porous, with clear differences between primary and secondary growth of stem.

Our study of *Heptacodium miconioides* provides first information on its phenology and anatomy structure and hints what could be investigated in future experiments. Further research could lead to deeper understanding of climate-growth and phenology relationship, and in studies of meaning of cambium activity in late-flowering woody plants. It could also lead to acquiring further knowledge on the mechanism of adaptation of woody plants flowering into late autumn to different climate conditions distinct from their natural habitat. Additionally, such research could deepen our understanding of potential chances of survival of endangered species outside their normal distribution’s area. Also, such further research can focus on identification of metabolites found in the stem at various phenological phases and screening for those with useful properties, or simply their general chemoprofiling. It could be especially interesting, as *Heptacodium* is notable for lack of diseases and pests infecting the bushes, which suggests presence of very interesting protective compounds in the plant.

The research indicated the adaptive potential of *Heptacodium* in response to climatic condition of temperate zone.

## Data Availability

All relevant data supporting the findings of this study can be found in the manuscript and supplementary materials and are available from the corresponding author upon reasonable request.

## References

[1] Yang Q, Landrein S, Osborne J, Borosova R, Wu ZY, Raven PH, Hong DY (2011). Caprifoliaceae. Flora of China. Vol. 19. Cucurbitaceae through Valerianaceae, with Annonaceae and Berberidaceae.

[2] Missouri BG. *Heptacodium miconioides*. 2022. http://www.missouribotanicalgardeNorg/PlantFinder/PlantFinderDetailSaspx?kempercode=k450. Available March 13, 2022.

[3] Flora of China. *Heptacodium miconioides* Rehd. 2023. http://www.efloras.org/florataxon.aspx?flora_id=2&taxon_id=200022268. Available March 16, 2023.

[4] Lu HP, Cai YW, Chen XY, Zhang X, Gu YJ, Zhang GF (2006). High RAPD but no cpDNA sequence variation in the endemic and endangered plant, *Heptacodium miconioides* Rehd. (*Caprifoliaceae*). Genetica.

[5] The IUCN red list of threatened species. Available from https://www.iucnredlist.org/species/32355/9700631. Cited 2021 Dec 30.

[6] Bian C, Jin ZX, Li J (2002). A study on the reproductive biology of *Heptacodium miconioides*. Acta Bot Yunnanica.

[7] Jin ZX (1998). A study on *Heptacodium miconioides* community in the Tiantai Mountains of Zhejiang province. Acta Ecol Sin.

[8] Jin ZX (1997). A study on population structure and distribution pattern of *Heptacodium miconioides* in the Tiantai Mountain, Zhejiang. Chin J Ecol.

[9] Jin ZX (2002). A study of dominant population structure and interspecific association of *Heptacodium miconioides* community in Tiantai Mountain of Zhejiang province. Bull Bot Res.

[10] Li J, Jin ZX (2006). Genetic diversity of natural *Hepatacodium miconioides* populations in Zhejiang province. Front For China.

[11] Jin ZX, Li J (2006). Anti-bacterial activity of leaf extracts from *Heptacodium miconioides*. J Zhejiang A&F Univ.

[12] Jin ZX, Li J (2006). Anti-bacterial activity of extracts from *Heptacodium miconioides*. J Zhejiang A&F Univ.

[13] Yang GB, Shao H, Jin ZX. Analysis of secondary metabolism contents in leafblades of *Heptacodium miconioides*. 2011. http://en.cnki.com.cn/Article_en/CJFDTOTAL-XBLX200602035.htm. Cited 30 12 2021.

[14] Jacobs B, Geuten K, Pyck N, Huysmans S, Jansen S, Smets E (2011). Unraveling the phylogeny of *Heptacodium* and *Zabelia* (*Caprifoliaceae*): an interdisciplinary approach. Syst Bot.

[15] RHS 2022. *Heptacodium miconioides*. https://www.rhs.org.uk/plants/75426/i-heptacodium-miconioides-i/details. Available March 13, 2022.

[16] Larcher F, Battisti L, Pomatto E, Devecchi M (2021). Woody species and supporting ecosystem services: the case study of the city of Turin (Italy). Acta Hortic.

[17] Karla G, Lal MA, Bhatla SC, Lal MA (2018). Physiology of flowering. Plant physiology, development and metabolism.

[18] Monder MJ, Woliński K, Niedzielski M, Pacholczak A (2016). The impact of seasonal changes in plant tissue on rhizogenesis of stem cuttings of the once flowering roses. Not Bot Horti Agrobot Cluj Napoca.

[19] Monder MJ, Kozakiewicz P, Jankowska A (2017). Effect of anatomical structure of shoots in different flowering phase on rhizogenesis of once-blooming roses. Not Bot Horti Agrobot Cluj Napoca.

[20] Monder MJ, Kozakiewicz P, Jankowska A (2019). Anatomical structure changes in stem cuttings of rambler roses induced with plant origin preparations. Sci Hort.

[21] Monder MJ, Kozakiewicz P, Jankowska A (2021). The role of plant origin preparations and phenological stage in anatomy structure changes in the rhizogenesis of *Rosa* ‘Hurdal’. Front Plant Sci.

[22] Aloni R, Raghavendra AS (1991). Wood formation in deciduous hardwood trees. Physiology of trees.

[23] Čufar K, Cherubini M, Gričar J, Prislan P, Spina S, Romagnoli M (2011). Xylem and phloem formation in chestnut (*Castanea sativa* Mill.) during the 2008 growing season. Dendrochronologia.

[24] Patel VR, Pramod S, Rao KS (2014). Cambial activity, annual rhythm of xylem production in relation to phenology and climatic factors and lignification pattern during xylogenesis in drum-stick tree (*Moringa oleifera*). Flora.

[25] Morellato LPC, Alberton B, Alvarado ST, Borges B, Buisson E, Camargo MGG, Cancian LF, Carstensen DW, Escobar DFE, Leite PTP, Mendoza I, Rocha NMWB, Soares NC, Silva TSF, Staggemeier VG, Streher AS, Vargas BC, Peres CA (2016). Linking plant phenology to conservation biology. Biol Conserv.

[26] Rossie S, Morin H, Deslauriers A (2012). Causes and correlations in cambium phenology: towards an integrated framework of xylogenesis. J Exp Bot.

[27] Frankenstein C, Eckstein D, Schmitt U (2005). The onset of cambium activity — a matter of agreement?. Dendrochronologia.

[28] Donaldson LA (1992). Lignin distribution during latewood formation in *Pinus radiata* D. Don. IAWA Bull NS.

[29] Sass-Klassen U, Sabajo CR, Ouden J (2011). Vessel formation in relation to leaf phenology in pedunculate oak and European ash. Dendrochronologia.

[30] Soute-Herrero M, Rozas V, Garcīa-González I (2018). Early-wood vessels and latewood width explain the role climate on wood formation of *Querqus pyrenaica* Willd. across the Atlantic-Mediterranean boundary in NW Iberia. Forest Ecol Manag.

[31] Vitasse Y, François C, Delpierre N, Dufrêne E, Kremer A, Chuine I, Delzon S (2011). Assessing the effects of climate change on the phenology of European temperate trees. Agric Forest Meteorol.

[32] Satake A, Kawagoe T, Saburi Y, Chiba Y, Sakurai G, Kudoh H (2013). Forecasting flowering phenology under climate warming by modelling the regulatory dynamics of flowering-time genes. Nat Commun.

[33] Jabłońska K, Kwiatkowska-Falińska A, Czernecki B, Wawelender JP (2015). Changes in spring and summer phenology in Poland - responses of selected plant species to air temperature variations. Pol J Ecol.

[34] Monder MJ, Niedzielski M. Evaluation of frost resistance of rambler roses based on electrolytes leakage. Acta Hortic. 2021;1331:285-92. 10.17660/ActaHortic.2021.1331.38.

[35] Przybylak R, Majorowicz J, Wójcik G (2005). Temperature changes in Poland from the 16th to the 20th centuries. Int J Climatol.

[36] Niedzwiedź T, Limanówka D (1992). Termiczne pory roku w Polsce. Zesz Nauk UJ PR Geogr.

[37] Bąbelewski P (2014). Phenological phases of tree of heaven (*Ailanthus altissima* (Mill.) Swingle) in different use zones of the city Wrocław. Part 1: vegetative development phases. Zesz Nauk UP we Wrocławiu “Rolnictwo”.

[38] Bąbelewski P (2014). Phenological phases of tree of heaven (*Ailanthus altissima* (MilL) Swingle) in different use zones of the city Wrocław. Part 2: generative development phases. Zesz Nauk UP we Wrocławiu “Rolnictwo”.

[39] Krużel J, Ziernicka-Wojtaszek A, Borek Ł, Ostrowski K (2015). Zmiany czasu trwania meteorologicznego okresu wegetacyjnego w Polsce w latach 1971–2000 oraz 1981–2010. Ecol Eng.

[40] Graczyk D, Szwed M (2020). Changes in the occurrence of late spring frost in Poland. Agronomy.

[41] Liu Q, Piao S, Janssens IA, Fu Y, Peng S, Lian X, Ciais P, Myneni RB, Peñuelas J, Wang T (2018). Extension of the growing season increases vegetation exposure to frost. Nat Commun.

[42] Primack RB (2009). The role of botanical gardens in climate change research. New Phytol.

[43] Cleland EE, Chiariello NR, Loarie SR, Mooney HA, Field CB (2006). Diverse responses of phenology to global changes in a grassland ecosystem. P Natl Acad Sci USA.

[44] Plant Map 2022. Poland plant hardiness zone map 2020. Available November 19, 2022. https://www.plantmaps.com/interactive-poland-plant-hardiness-zone-map-celsius.php.

[45] Szymanowski M. Miejska wyspa ciepła we Wrocławiu. Wrocław: Wydawnictwo Uniwersytetu Wrocławskiego, Studia Geograficzne – tom 77; 2005.

[46] Dubicki A, Dubicka M, Szymanowski M, Smolnicki K, Szykasiuk M (2002). Klimat Wrocławia. Środowisko Wrocławia Informator o stanie środowiska Wrocławia.

[47] Szymanowski M. Modeling the urban heat island of Wrocław. In: Man and climate in 20th century. Wrocław: Doln. Wyd. Infor.. 2002. p. 89–90.

[48] Sudnik-Wójcikowska B. Flora miasta Warszawy i jej przemiany w ciągu XIX i XX wieku. Część I. Warszawa: Wydawnictwo Uniwersytetu Warszawskiego. 1987.

[49] Meteorological Data 2022. https://en.tutiempo.net/climate/12-2021/ws-123750.html. Available February 20, 2022.

[50] Bleiholder H, Buhr L, Feller C, Hack H, Hess M, Klose R, Lancashire BD, Meier U, Stauss R, Boom T, Weber E. Growth stages of mono-and dicotyledonous plants. BBCH monograph. In: Meier U, editor. Julius Kühn-Institut (JKI): Quedlinburg; 2018. p. 204.

[51] Meier U (1997). Growth stages of plants – Entwicklungsstadien von Pflanzen – Estadios de las plantas – Développement des Plantes. BBCH-Monograph.

[52] Kluza M, Zientarska A (1999). Obserwacje fenologiczne fazy kwitnienia wybranych gatunków krzewów w Ogrodzie Dendrologicznym Akademii Rolniczej w Poznaniu. Bibl Fragm Agron.

[53] Łukaszewicz A (1984). Potrzeba ujednolicenia metodyki fenologicznej w Polskich Ogrodach Botanicznych i Arboretach. Wiad Bot.

[54] Łukasiewicz S (1999). Modyfikacje metody wykreślania diagramów fenologicznych drzew rosnących w warunkach miejskich w oparciu o obserwacje *Aesculus hippocastanum* L. na terenie Poznania. Biuletyn Ogrodów Botanicznych.

[55] Bond J, Donaldson L, Hill S, Hitchcock K (2018). Safranin fluorescent staining of wood cell walls. Biotech Histochem.

[56] Jansen S, Kitin P, De Pauw H, Idris M, Beeckman H, Smets E (1998). Preparation of wood specimens for transmitted light microscopy and scanning electron microscopy. Belg J Bot.

[57] Gao J, Yu M, Zhu S, Zhou L, Liu S (2019). Effects of exogenous 24-spibrassinolide and brasinazole on negative gravitropism and tension wood formation in hybrid poplar (*Populus deltoides* x *Populus nigra*). Planta.

[58] Pelc S (1964). Wybrane zagadnienia z metodyki nauczania biologii: technika wykonywania botanicznych preparatów mikroskopowych. Rocznik Naukowo-Dydaktyczny WSP w Krakowie.

[59] Wheeler E, Baas P, Gasson P (1989). IAWA List of microscopic features for hardwood identification with appendix on non-anatomical information. IAWA Bull.

[60] Angyalossy V, Pace MR, Evert RF, Marcati CR, Oskolski AA, Terrazas T, Kotina E, Lens F, Mazzoni-Viveiros SC, Angeles G, Machado SR, Crivellaro A, Rao KS, Junikka L, Nikolaeva N, Baas P (2016). IAWA list of microscopic bark features. IAWA J.

[61] Wheller EA (1986). Vessels per square milimetre or vessel groups per square milimetre?. IAWA Bull.

[62] Kopcewicz J, Kopcewicz J, Lewak S (2002). Rozwój generatywny. Fizjologia roślin.

[63] Abbott DL, Luckwill LC, Cutting CV (1970). The role of bud scales in the morphogenesis and dormancy of the apple fruit bud. Physiology of tree crops.

[64] Hejnowicz Z (2012). Anatomia i histogeneza roślin naczyniowych. Organy wegetatywne.

[65] Karlman L, Morling T, Martinsson O (2005). Wood density, annual ring width and latewood content in larch and scots pine. Eurasian For Res.

[66] Carrillo I, Aguayo MG, Mendonça SVRT, Elissethe JP (2005). Variations in wood anatomy and fiber biometry of *Eucalyptus globulus* genotypes with different wood density. Wood Res.

[67] Barotto JA, Monteoliva S, Gyenge J, Martínez-Meier A, Moreno K, Tesón N, Fernández ME (2017). Wood density and anatomy of three Eucalyptus species: implications for hydraulic conductivity. For Syst.

